# Pre-Micro RNA Signatures Delineate Stages of Endothelial Cell Transformation in Kaposi Sarcoma

**DOI:** 10.1371/journal.ppat.1000389

**Published:** 2009-04-17

**Authors:** Andrea J. O'Hara, Pauline Chugh, Ling Wang, Eduardo M. Netto, Estrella Luz, William J. Harrington, Bruce J. Dezube, Blossom Damania, Dirk P. Dittmer

**Affiliations:** 1 Department of Microbiology and Immunology, Lineberger Comprehensive Cancer Center, Center for AIDS Research at the University of North Carolina at Chapel Hill, Chapel Hill, North Carolina, United States of America; 2 Federal University of Bahia, Salvador, Bahia, Brazil; 3 The Viral Oncology Program, Sylvester Comprehensive Cancer Center, University of Miami Miller School of Medicine, Miami, Florida, United States of America; 4 Division of Hematology/Oncology, Beth Israel Deaconess Medical Center, Harvard Medical School, Boston, Massachusetts, United States of America; Oregon Health and Science University, United States of America

## Abstract

MicroRNAs (miRNA) have emerged as key regulators of cell lineage differentiation and cancer. We used precursor miRNA profiling by a novel real-time QPCR method (i) to define progressive stages of endothelial cell transformation cumulating in Kaposi sarcoma (KS) and (ii) to identify specific miRNAs that serve as biomarkers for tumor progression. We were able to compare primary patient biopsies to well-established culture and mouse tumor models. Loss of mir-221 and gain of mir-15 expression demarked the transition from merely immortalized to fully tumorigenic endothelial cells. Mir-140 and Kaposi sarcoma–associated herpesvirus viral miRNAs increased linearly with the degree of transformation. Mir-24 emerged as a biomarker specific for KS.

## Author Summary

MicroRNAs are key regulators of cancer and development. We can use their pattern of expression to classify different cancers or in our case different stages in cancer development. We used a novel method to define progressive stages for Kaposi sarcoma (KS), which is a cancer of the endothelial cells. We identified specific precursor-miRNAs that accurately identify stages of KS tumor progression. For the first time, we were able to profile KS patient material. This is difficult to come by, but it is more closely related to the human cancer than even the best cell culture models. Our work statistically defined clusters of pre-miRNAs, each signifying one step in cancer progression. This is the first time that precursor miRNAs profiling was used to define cancer stages. It is also the first time that we have defined makers of any kind that allow us to distinguish between different types of the endothelial cancer KS. Loss of mir-221 precursor miRNA and gain of mir-15 precursor miRNA expression demarked the transition from merely immortal to fully tumorigenic cells. Mir-140 and Kaposi sarcoma–associated herpesvirus viral microRNAs increased progressively with the degree of transformation, i.e. more aggressive stages expressed higher levels of these biomarkers. High levels of the precursor microRNA mir-24 emerged as a biomarker only in patient derived KS samples, not in any of the culture models.

## Introduction

Kaposi sarcoma (KS) is one of the few human cancers of endothelial origin. KS remains the most frequent AIDS-associated malignancy even in populations with ready access to highly active anti-retroviral therapy (HAART) [1],[2]. Today, approximately one third of AIDS-KS tumors develop in patients on successful long-term HAART, i.e. with near normal T lymphocyte counts and undetectable HIV viral loads [3]. In sub-Saharan Africa, KS ranks among the most common cancers overall since HIV turned the endemic form of this disease into an epidemic. By comparison to epithelial cancers, endothelial-lineage cancers are less common still, and most study endothelial cells because of their ancillary role in tumor angiogenesis rather than their role as the driving force of tumor formation. In KS, endothelial lineage cells drive tumor growth. Recent data suggest that tumor-associated stromal cells, including endothelial cells can acquire epigenetic or perhaps even genetic features of transformation, which in turn support tumor growth [4],[5],[6]. KS offers the opportunity to study endothelial cell transformation and tumorigenesis in detail, and miRNAs provide one possible means of large-scale, stable epigenetic reprogramming. To test the hypothesis that miRNA signatures delineate progressive stages of endothelial cell transformation resulting in metastatic KS, we used high throughput, quantitative real-time PCR-based pre-miRNA profiling.

KS is tightly associated with Kaposi sarcoma associated herpesvirus (KSHV) [7],[8],[9]. Every tumor cell carries the virus and expresses at least the viral latent proteins [10],[11]. Here, we show for the first time using primary patient biopsies that every KS tumor transcribes the viral miRNAs (miRNA) as well.

KSHV is also the etiological agent of the B cell lineage tumor primary effusion lymphoma (PEL), as well as the B cell lineage hyperplasia, plasmablastic variant of multicentric Castleman disease (MCD) [12],[13]. This dichotomous phenotype (lymphoid/endothelial) allows testing of the hypotheses that the miRNA profile for these two cancers reflect (a) their tissue of origin, (b) progressive cancer signatures, (c) a signature induced by latent viral infection or (d) a combination of all.

The miRNAs have emerged as master regulators of cell lineage differentiation and key modulators of cancer (reviewed in [14]). They are small, 22 nucleotide non-coding RNA molecules that, upon incorporation into the cytoplasmic RNA-induced silencing complex (RISC), can inhibit translation of target messenger RNAs and ultimately target them for degradation. At present, the Sanger database has recorded 678 human miRNAs [15] each capable of targeting up to several hundred different mRNAs. KSHV encodes multiple viral miRNAs [16],[17],[18],[19], including a viral ortholog to miR-155 [20],[21]. Even though some targets for these viral miRNAs have been identified [22], exactly how they function in KS tumorigenesis is unresolved.

MiRNA profiling has provided invaluable insights into tissue development and cancer. Many tumor-specific and cell lineage-specific signatures have been compiled (e.g. [23],[24],[25] and many others). We previously established the miRNA signature for PEL [26] using real-time quantitative PCR. Pre-miRNA profiling has also been used successfully to stratify human tumors. It often correlates well with mature miRNA levels [27],[28],[29], but we also found that pre-miRNA profiling provides non-redundant information with utility for tumor classification. Pre-miRNAs are an intermediate product for mature miRNAs, analogous—in the widest sense—to mRNAs being an intermediate product for proteins. They are generated by Drosher and DGCR8 from the nascent pri-miRNAs and eventually exported by Exportin-5 to the cytoplasm. For the purpose of profiling they offer advantages because they are longer (∼70 nt), each nucleotide contributing additional specificity in diagnostic assay, as opposed to mature miRNAs, which because of their limited target region of only ∼22 nt impose limitations due to cross hybridization and variable primer/probe annealing efficiency for different miRNAs. Here, we use real-time QPCR-based profiling to discern pre-miRNAs that identify KS, KSHV infection and distinct, progressive stages of endothelial cell transformation.

KSHV transforms primary human endothelial cells in culture [30], though this is a rare event. KSHV infection consistently only leads to morphological alterations (“spindling”) and reduced growth factor dependence [31],[32],[33],[34]. KSHV infection of immortalized human endothelial cells leads to extended survival, and growth factor independence [35],[36],[37] but not complete transformation, as defined by the ability to form tumors in nude mice. Importantly, during latent viral infection, lymphatic endothelial cell differentiation markers remain expressed, and if not already of lymphatic endothelial origin, KSHV is capable of inducing this phenotype in human endothelial cell preparations derived from other tissues such as the vasculature [35],[38],[39]. KSHV infection per se does not induce dedifferentiation of lineage-committed lymphatic endothelial cells. A study by An et al. succeeded in deriving two fully tumorigenic clones of lymphatic endothelial cells (TIVE E1 and TIVE L1) that maintain KSHV in the absence of selection [40]. Introduction of KSHV into murine endothelial progenitor cell preparations also resulted in the clonal outgrowth of at least one fully transformed cell line [41]. By contrast, attempts to culture tumor cells directly from KS lesions largely failed. Today, we have only a single KS tumor derived cell line, SLK, which is fully transformed, but has lost the KSHV genome [42]. Together with primary KS biopsies, these culture systems exemplify multiple stages of endothelial cell cancer progression (Table 1). In this study, we used high throughput profiling to identify cell lineage and cancer progression stage-specific pre-miRNAs for this representative set of KSHV-infected and uninfected immortalized endothelial cells, KS biopsies and PEL lymphoma cell lines.

**Table 1:** Samples representative of different stages in endothelial cell transformation

SAMPLES	KSHV	TUMOR^a^	REFERENCE	STAGE^e^
**Cell lines:**				
tertHUVEC	-	-	b	E
tertHMVEC	-	-	b	E
tertHUVEC-2	+	-	b	EK
tertHUVEC-6	+	-	b	EK
tertHMVEC-1	+	-	b	EK
E1 TIVE	+	+	c	ET
L1 TIVE	+	+	c	ET
SLK	-	+	d	ET
E1 Tumor-1	+	++	c	ETM
E1 Tumor-2	+	++	c	ETM
E1 Tumor-3	+	++	c	ETM
E1 Tumor-4	+	++	c	ETM
E1 Tumor-5	+	++	c	ETM
**Biopsies:**				
KS-1	+	+++	this study	KS
KS-2	+	+++	this study	KS
KS-3	+	+++	this study	KS
KS-4	+	+++	this study	KS
KS-5	+	+++	this study	KS
KS-6	+	+++	this study	KS
KS-7	+	+++	this study	KS
KS-8 (2007)	+	+++	this study	KS
KS-8 (2008)	+	+++	this study	KS

a. Relative tumorigenicity: + tumor-forming capability in xenograft models; ++ tumor in matrigel support in mice at 5×105 cell; +++ KS tumor in a person.

b. Wang L, Damania B (2008) Kaposi sarcoma-associated herpesvirus confers a survival advantage to endothelial cells. Cancer Res 68: 4640–4648.

c. An, F.Q., et al., Long-term-infected telomerase-immortalized endothelial cells: a model for Kaposi sarcoma-associated herpesvirus latency in vitro and in vivo. J Virol. 2006. 80(10): p. 4833–46.

d. Herndier BG, et al. (1994) Characterization of a human Kaposi sarcoma cell line that induces angiogenic tumors in animals. AIDS 8: 575–581.

e. Stage identifies biological distinct classes of transformation: uninfected endothelial cells (E), KSHV-infected endothelial cells (EK), endothelial cells that have the ability to form tumors in nude mice (ET, which includes the KSHV-positive TIVE- E1, L1 cell lines as well as KSHV-negative SLK cells), xenograft tumors of TIVE- E1 cells consisting of 5 independent samples (ETM), KS patient biopsies (KS).

## Results

### Experimental Approach

A total of 47 samples were selected for profiling at the DNA and pre-miRNA level (Table 1 and Table S1). These include the largest number of PEL cell lines to date (n = 14). Four KSHV-negative Burkitt lymphoma cell lines were included as controls as they are expected to transcribe miRNAs that are common to B lineage lymphomas. The pre-miRNA difference between these samples and PEL defines part of the PEL signature [26]. For this study, we added 9 tonsil tissues as a normal tissue control. These serve to determine which pre-miRNAs are highly abundant in B cells, as tonsils consist of over 50% B cells, including germinal center (GC) B cells, which many assume to be the normal precursor of PEL [43],[44],[45]. Two T-cell lymphoma cell lines were included to differentiate T cell pre-miRNAs.

For the first time, KS primary biopsies were also assessed for pre-miRNA transcription. We collected 9 AIDS-KS skin biopsies from the Americas. The biopsies were collected by individuals with experience in KS clinical trials. They are considered representative lesions for the purpose of tumor and response staging. The majority of cells in each biopsy are KSHV-infected endothelial cells [11],[46],[47]. All biopsies were from male subjects with a median age of 44 years (range 30–57). Patients had biopsy-confirmed KS and were on HAART as well as concurrent chemotherapy. Their median CD4 count was 78 cells/microliter (range 7–402). CD4 counts were not available for one subject. All patients had extensive cutaneous KS and with the exception of one are alive at present. Tumor samples were obtained within the last three years. Hence, these patients represent the current post-HAART AIDS epidemic.

Two immortalized virus-negative endothelial cell lines were included in the arrays, as well as isogenic controls carrying latent KSHV [36]. A similar model exists in the E1 TIVE and L1 TIVE cell lines [40]. These currently represent the best human cell culture tumor model for KS, as these two cell lines induce KS-like tumors in nude mice with 100% efficiency. Also included is the only known KS-derived cell line, SLK [42], which has lost the KSHV genome, but is tumorigenic in mice.

As positive control we used DNA as the input and real-time QPCR and primers directed against the pre-miRNA as described [26]. We were able to independently verify KSHV-infection status for each sample. Likewise, the EBV miRNA genes were detectable only in the EBV-positive PEL and BL cell lines, but not KS or any other samples. EBV miRNA genes were not detectable in normal tonsil tissue. The relative copy number for the KSHV miRNA genes was significantly lower in KSHV carrying endothelial cell lines compared to PEL (see Figure S4). This is consistent with earlier reports that PEL carry more viral plasmids (50∼100 copies/cell) than KS (∼10 copies/cell) and KSHV-infected endothelial cell cultures [46],[48],[49],[50]. Among the KSHV infected endothelial cell models, the HMVEC carried the highest KSHV genome number, suggesting that they are most capable of maintaining high levels of the KSHV plasmid. This is consistent with earlier studies showing that not all endothelial cells are equally permissive for KSHV infection, which drives reprogramming towards lymphatic endothelial cells [35],[38],[39].

### Unsupervised clustering reveals the pre-miRNA profile of KS

Our pre-miRNA data set, which included 160 primer pairs, representing 145 cellular miRNAs, 9 viral miRNAs, 2 viral mRNAs and 4 cellular RNAs (U6), and 47 samples consisted of >20,000 individual data points. QPCR measures target abundance on a _2_log scale with higher CT numbers reflecting lower abundance. For this analysis, the average of the triplicate CT values was taken. These were normalized to U6 levels, to give dCT. Note that dCT values represent the underlying pre-miRNA levels on a _2_log scale thus facilitating robust clustering [51],[52]. Following normalization, each sample set was Z-standardized to remove variation between samples [53],[54].

**Figure 1:**
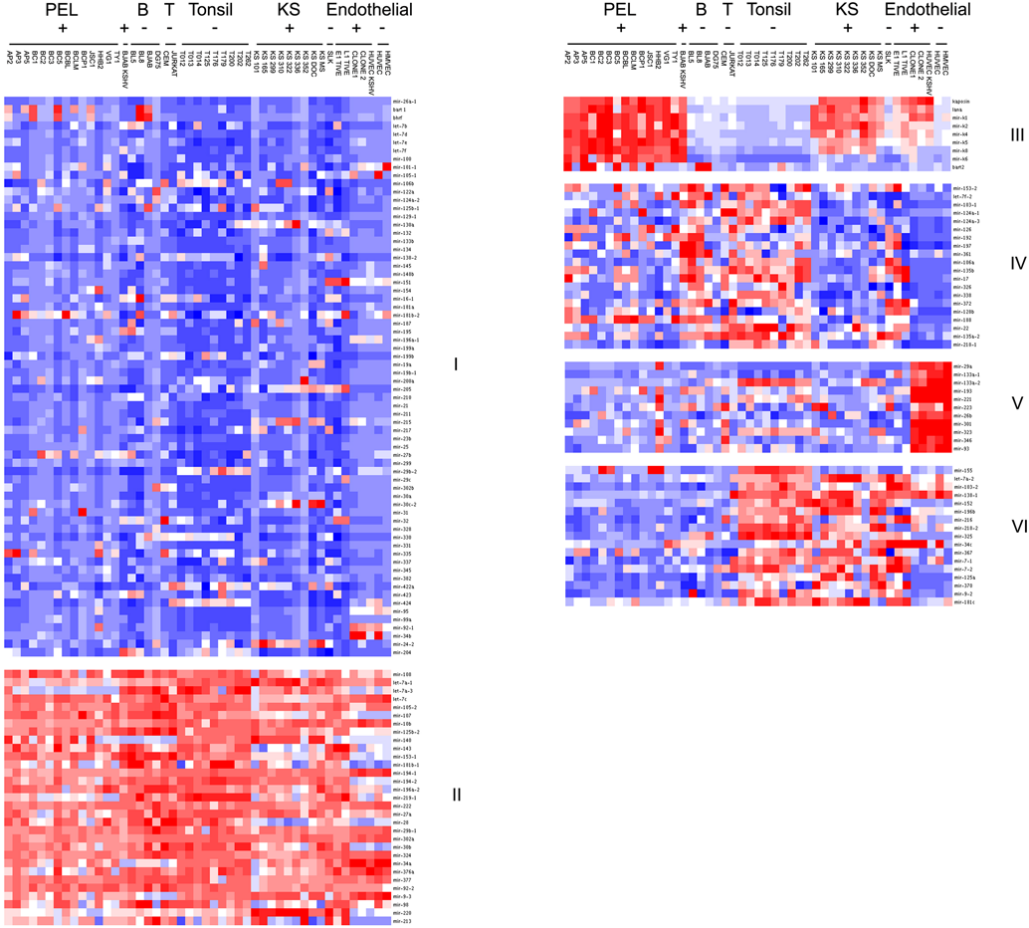
Pre-miRNA profiling of all samples. Shown is a heatmap, with blue indicating low levels of signal, white intermediate, and red high levels of signal, when comparing across all primer pairs. The horizontal axis indicates samples: PEL indicates PEL, B indicates KSHV-negative B cell lymphoma, T indicates T cell lymphoma, Tonsil indicates tonsil and Endothelial indicates all endothelial culture cell models, including SLK, E1 and L1 TIVE, and infected and uninfected HUVEC and HMVEC cells. Additionally, viral status is shown, with + indicating KSHV-positive and – indicating KSHV-negative. The vertical axis indicates the pre-miRNA. Groups I and II represent the miRNAs that are unchanged across all samples. Group III indicates cellular and KSHV miRNAs induced upon KSHV infection. Group IV represents the miRNAs that are downregulated in KSHV-positive infection. Group V represents 11 cellular miRNAs highly expressed in the HUVEC and HMVEC cells, both uninfected and infected. Group VI contains cellular miRNAs that are downregulated in all B-cell lymphomas, including PEL.

Figure 1 shows the heatmap representation after hierarchical clustering for the full panel of samples, with red indicating a higher level of expression and blue indicating a lower level of expression compared to the median of all data (white). 6 distinct groups were identified. These represent the minimal number of non-overlapping clusters based on principal component analysis (PCA) (data not shown). The first two groups represent the pre-miRNAs that are unchanged across all samples, those with low levels of expression (I in blue) and those with high levels of expression across all samples (II in red). The KSHV pre-miRNAs all cluster in group III. Group IV represents the pre-miRNAs that are downregulated in KSHV-positive cells. 20 miRNAs are contained in this group. Group V represents 11 cellular miRNAs that are highly expressed in immortalized HUVEC and HMVEC cells, both uninfected and KSHV-infected, but not any of the tumor cell lines and biopsies. They do not appear to be significantly enriched in any of the other endothelial cell types (KS or TIVE). Finally, group VI contains cellular miRNAs that are downregulated in all B-cell lymphomas, including PEL, vis-à-vis tonsil and KS.

**Figure 2:**
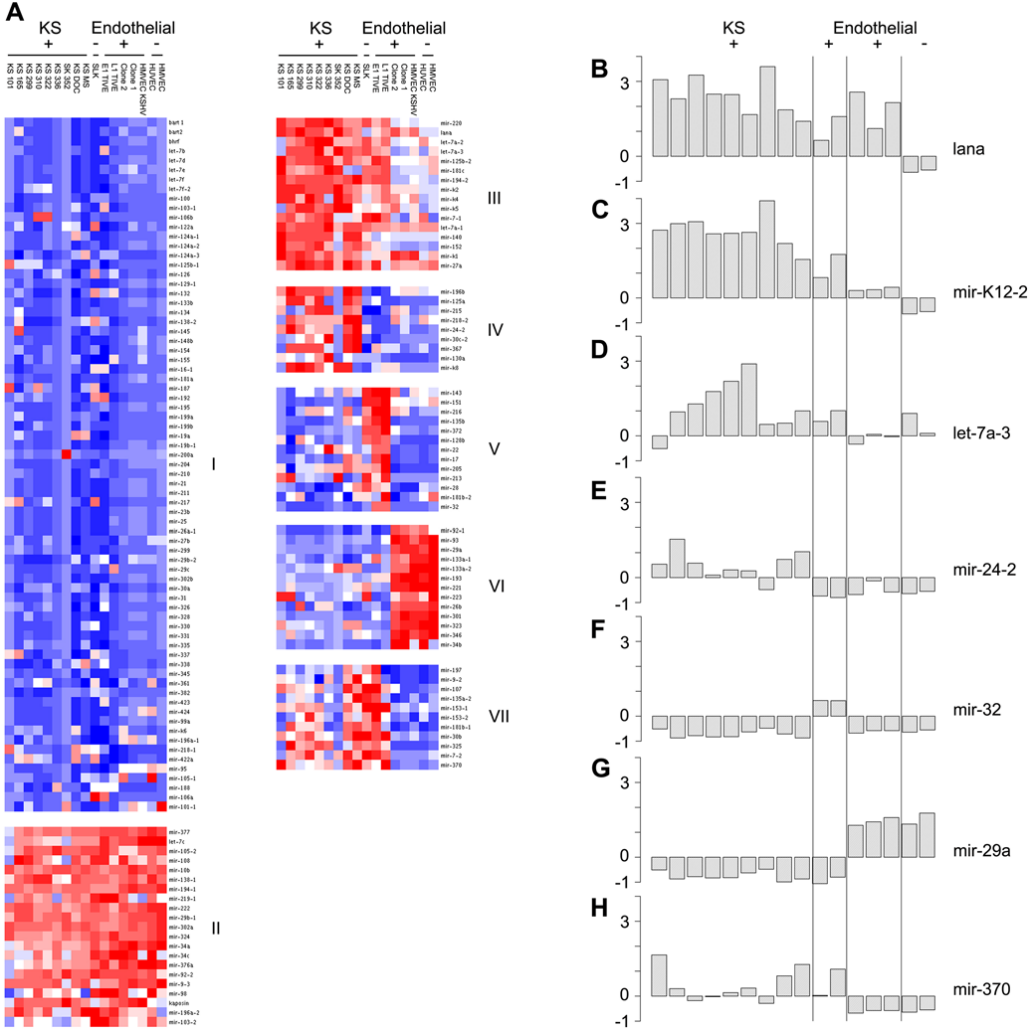
Pre-miRNA profiling of primary KS biopsies and endothelial derived cell cultures. (A) Shown is a heatmap, with blue indicating low levels of signal, white intermediate, and red high levels of signal, when comparing across all primer pairs. The horizontal axis indicates samples: KS indicates primary KS biopsies and Endothelial indicates all endothelial culture cell models, including SLK, E1 and L1 TIVE, and infected and uninfected HUVEC and HMVEC cells. Additionally, viral status is shown, with + indicating KSHV-positive and – indicating KSHV-negative. The vertical axis indicates the pre-miRNA. Group I and II represent the miRNAs that are unchanged in all samples. Group III shows the cellular and viral miRNAs that are upregulated upon KSHV infection. Group IV indicates viral and cellular miRNAs upregulated specifically in KS, group V for E1 and L1 TIVE, and group VI for uninfected and infected HUVEC and HMVEC cells. Group VII are cellular miRNAs downregulated in uninfected and infected HUVEC and HMVEC cells. Bar graphs representative of each group described are shown on the left (B–H). All samples are shown in the same order as the clustering, with KS representing primary KS biopsies and Endothelial indicating all endothelial cell culture models profiled. Again, viral status is also listed. In the bar graphs, each cell type is divided by a black bar. All bar graphs are dCT U6 normalized and Z-standardized. (B) Lana expression is high in all KSHV-positive cell lines and not present in KSHV-negative cell lines. (C) KSHV-mir-K2 is upregulated in all KSHV-positive samples, with the highest level of expression in primary KS biopsies, intermediate in E1 and L1 TIVE cells and lowest in infected HUVEC and HMVEC cells. (D) Cellular miRNA let-7a-3 shows a similar expression pattern of increased expression upon KSHV infection. (E) Mir-24-2 has increased expression in primary KS biopsies, as compared to other cell types. (F) Mir-32 has increased expression in E1 and L1 TIVE cells, as compared to other cell types. (G) Mir-29a has increased expression in both infected and uninfected HUVEC and HMVEC cells while mir-370 (H) shows reduced expression in this group, as compared to other cell types.

To remove the impact of lineage-specific determinants [B cell (PEL and Tonsil) vs. endothelial cell] from the analysis, we analyzed the two KSHV-associated cell types separately. Our analysis of PEL specific miRNAs was previously published [26] and analysis of the extended data set confirmed this observation (data not shown). When the endothelial-derived subset of samples was analyzed alone, a clearer picture emerged that highlights similarities and disparities between different stages of endothelial cell transformation (Figure 2A). The groups represent the minimal number of non-overlapping clusters based on PCA (data not shown). The first two groups (I and II) represent miRNAs with minimal discernable patterns across all samples—at least at the power of our analysis. Blue indicates low levels while red indicates comparable high levels of miRNAs, vis-à-vis the median of all data in this set. This is not to say that pre-miRNAs within these two clusters did not exhibit any change between samples classes, only that these changes were smaller compared to others and therefore less interesting from a biomarker perspective. For example, mir-222 clusters in group II because it was more highly transcribed in all samples relative to 50% of all other pre-miRNAs. Nevertheless mir-222 was downregulated in KSHV-infected, tumorigenic samples, compared to EC. The pattern of mir-222 parallels that of mir-221, which is expected because of their known co-regulation [55],[56]. However, the range of change was much larger for mir-221 as seen in group IV.

Group III shows the pre-miRNAs that are upregulated upon KSHV infection of EC and increased even more in KS and in the tumorigenic L1/E1 clones. This group includes the KSHV pre-miRNAs along with the cellular pre-miRNAs let-7a-1, let-7a-2, let-7a-3, mir-7-1, mir-27a, mir-125b-2, mir-140, mir-152, mir-181c, mir-194-2, and mir-220. The detailed transcription pattern for this group is shown as a bar graph for pre-miRNA let-7a-3 (Figures 2D, all bar graphs show Z-standardized values of median dCT_U6_).

To demark the degree of viral latent transcription, LANA mRNA levels are shown (Figure 2B). LANA is transcribed in all KSHV-positive samples but not the KSHV-negative SLK, HUVEC or HMVEC cell lines. KSHV latent RNA levels correlated positively with increasing tumor-forming capability of the infected cells (p≤10^−13^ by ANOVA of linear model). They were undetectable in uninfected cells, lowest in KSHV-infected HUVEC and E1/L1 cells in culture, higher in E1 mouse tumors and KS lesions and highest in PEL (data not shown). This was mirrored by KSHV pre-mir-K12-2 (Figure 2C). KSHV pre-miRNA transcription levels correlated KSHV plasmid copy number (DNA) as measured by real-time QPCR using the same primer sets with DNA as input (data not shown). The positive correlation between the level of viral miRNA and the relative tumorigenicity of the sample class supports a causal role for miRNA in KS tumorigenesis. It suggests that KSHV miRNAs are required to maintain the KS tumor phenotype. Group IV contains a set of 8 cellular miRNAs that are highest expressed in KS tumors only, compared to cell lines. These include mir-24-2, mir-30c-2, mir-125a, mir-130a, mir-196, mir-215, mir-218-2, and mir-367. The bar graph of mir-24-2 levels in Figure 2E serves as an example for the pre-miRNA expression pattern of this group, for which miRNA levels were highest in KS tumors and significantly lower in other samples whether KSHV-infected or not. As expected for all primary tumor samples, we observed more heterogeneity in the KS biopsies compared to clonal cell lines. This necessitated the use of 9 independent biopsies, which is a larger number then used in prior KS mRNA array analyses. With this number of biopsies, PCA analysis validated the significance of cluster membership for all pre-miRNA, including those that group in cluster IV.

Group V compromises a group of 13 cellular pre-miRNAs with highest levels in the E1 and L1 TIVE cell lines. These pre-miRNAs were present at higher levels in E1/L1 cells even compared even to KS biopsies. These are mir-17, mir-22, mir-28, mir-32, mir-128b, mir-135b, mir-143, mir-151, mir-181b-2, mir-205, mir-213, mir-216 and mir-372. The bar graph of mir-32 expression in Figure 2F is an example of the pre-miRNA expression pattern for this group.

Group VI consists of 13 pre-miRNAs with highest levels in the non-tumorigenic endothelial HUVEC and HMVEC cell lines, whether KSHV-infected or not. These are mir-26b, mir-29a, mir-34b, mir-92-1, mir-93, mir-133a-1, mir-133a-2, mir-193, mir-221, mir-223, mir-301, mir-323 and mir-346. 11 of these miRNAs were also contained in the HUVEC/HMVEC upregulated cluster from the larger data set (Figure 1). Additionally, mir-34b and mir-92-1 fell into this group upon clustering of only the endothelial cell data. The histogram of mir-29a expression in Figure 2G is an example of the pre-miRNA transcription pattern for this group, with highest levels in both infected and uninfected HUVEC/HMVEC cells and significantly lower levels in all other samples.

Group VII is the inverse of group VI and consists of miRNAs with undetectable levels in the endothelial HUVEC and HMVEC, whether KSHV-infected or not. This group compromises 11 cellular pre-miRNAs: mir-7-2, mir-9-2, mir-30b, mir-107, mir-135a-2, mir-153-1, mir-153-2, mir-181b-2, mir-197, mir-325 and mir-370. The bar graph of mir-370 expression in Figure 2H is an example of the pre-miRNA transcription pattern of this group.

In sum, unsupervised clustering as a discovery tool identified (i) distinct stages of endothelial cell transformation and (ii) specific pre-miRNAs that serve as biomarkers for each of them.

One of the concerns in profiling cell lines in culture is that the transcription signature may be reflective of a particular proliferation state rather than a general characteristic of the tumor subtype. Proliferation dependence is well documented for mRNA levels in fibroblasts [57]. For several miRNAs, too, proliferation and miRNA transcription rates are linked [58],[59],[60],[61],[62],[63],[64]. To guard against this fallacy, we only used RNA derived from log-phase cells for our profiling analysis. Nevertheless, to test the hypothesis that some miRNA levels were proliferation state dependent, we conducted a time course experiment for the E1 and L1 TIVE cell lines (see Figures S1 and S2). This revealed a very limited number of pre-miRNAs that were enriched in log-phase cells compared to stationary phase cells and vice versa. They were at the lower limit of detection and additional experiments are needed to validate the biological significance of this observation.

### miRNAs as endothelial cell tumor stage biomarkers

Unsupervised comparisons represent the first level of large scale profiling studies. Here, they revealed (i) the existence of multiple distinct steps of endothelial cell transformation and (ii) pre-miRNAs that were selectively transcribed in one or more stages and that therefore serve as biomarkers. The latter were further validated by supervised class prediction methods. Based upon pre-miRNA clustering (Figure 1 and 2) and published phenotype (Table 1), we defined the following classes: Endothelial cells (E), KSHV-infected endothelial cells (EK), endothelial cells that have the ability to form tumors in nude mice (ET), which includes the KSHV-positive TIVE- E1, L1 cell lines as well as KSHV-negative SLK cells), xenograft tumors of TIVE- E1 cells consisting of 5 independent samples (ETM), KS patient biopsies (KS), PEL (P), and as negative controls tonsil (TN) and non-KSHV associated lymphomas (TM).

First, we conducted pair-wise comparisons between classes using the median dCT_U6_ for each class (Figure S2). The two TERT-immortalized EC cell lines HUVEC and HMVEC exhibited a nearly identical pre-miRNA transcription pattern (r^2^ = 0.7238). Infection with KSHV of these immortalized cell lines did result in changes (r^2^ = 0.6798). Of note, this comparison is between median levels for the two EC cell lines (HUVEC and HMVEC) and three independent clones of tightly latently infected TERT-HUVEC cells. Thus, it exhibited more variability than a pair-wise comparison of just two cell lines. The most drastic change in overall pre-miRNA transcription emerged when comparing KSHV-infected, non-tumorigenic EC cell lines to the two KSHV-infected, highly tumorigenic E1/L1 cell lines. Here, we failed to detect any linear correlation. The two TIVE cell lines E1 and L1, of course, exhibited a strikingly similar pattern of pre-miRNA transcription as shown in detail in Figure S4 and Figure S1. The pair-wise comparison between E1/L1 cells in culture to E1 xenograft tumors showed a reasonable linear correlation, but less than between different culture models (r^2^ = 0.5684). Analysis of residuals identified all KSHV pre-miRNAs as well as mir-223 to be significantly upregulated in the tumorgraft (data not shown). Since there are no human infiltrating lymphocytes in the SCID mouse model, and since the tumor vasculature is made of murine endothelial cells, any changes in pre-miRNA composition reflect the grafted human tumor cells. Importantly, the comparison between E1 xenograft tumor biopsies and patient KS biopsies yielded a better correlation (r^2^ = 0.5846) than between E1/L1 cells in culture and E1 tumor grafts. This reinforces the results of the phenotypic characterization of E1/L1 cells [40] and demonstrates that the E1/L1 xenograft model adequately mimics primary KS patient biopsies.

**Figure 3:**
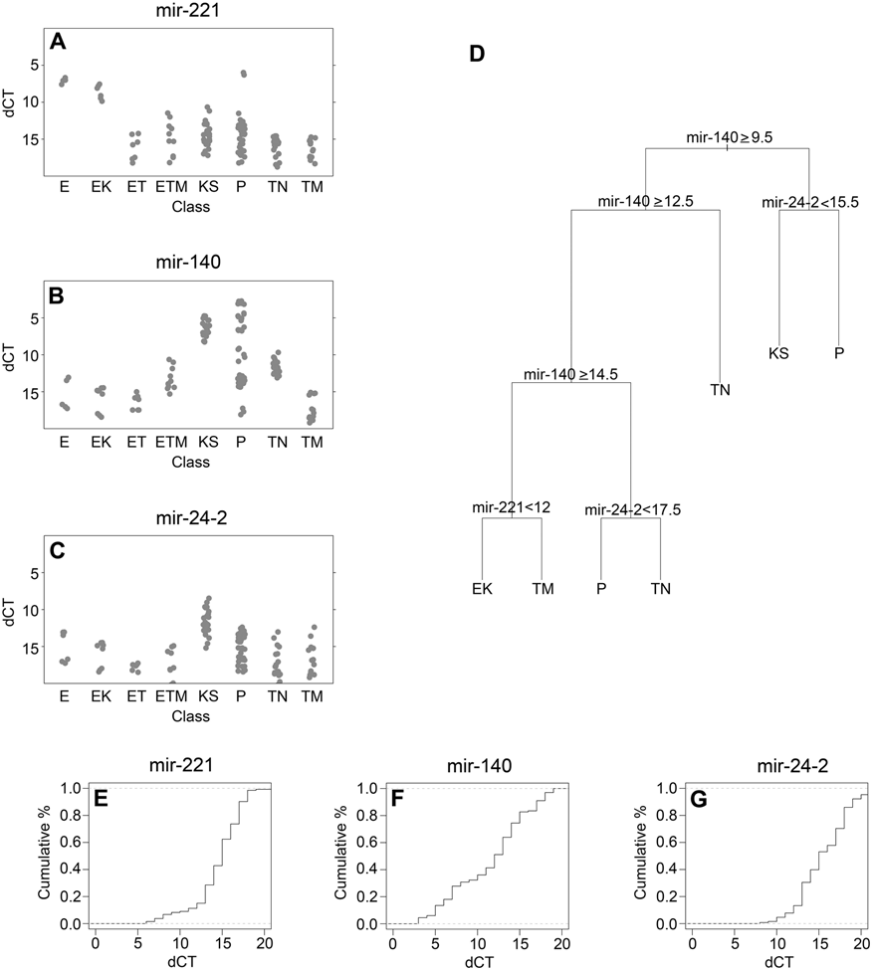
Validation of 4 individual biomarkers for endothelial cell transformation. (A–C) Shown are relative transcription levels (dCT) on the vertical and sample class on the horizontal axis. Dots indicate individual data points (>2 for each sample) for mir-221, mir-140, mir-24-2. (D) Decision tree of clustering based on just these 3 biomarkers. (E–G) Densityplots of dCT values for these four genes. Relative frequency (in %) is shown on the vertical axis and relative levels (dCT) on the horizontal axis. The dotted line indicates the cut-off value for class determination.

Next, we identified and validated a set of diagnostic pre-miRNA biomarkers that signify the different steps of endothelial cell transformation. To do so we used the miRNAs identified by hierarchical clustering (Figure 2), extended the dataset to include mouse xenograft tumor samples and used visual inspection followed by ANOVA and appropriate pair-wise t-test to identify pre-miRNAs with distinct distributions among the different steps of endothelial cell transformation. To give a better impression of within class variability, Figure 3 A–C plots individual dCT_U6_ for cellular pre-miRNAs including technical replicates for each class.

**Figure 4:**
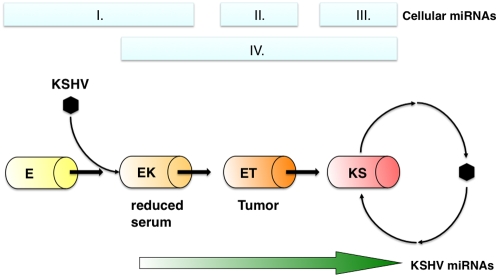
Model of KSHV-dependent progressive transformation of endothelial cells as identified by pre-miRNA clustering. Upon KSHV infection, KSHV miRNAs and cellular miRNAs are induced. Through various stages of transformation, additional cellular miRNAs are induced and KSHV miRNA expression increases. E indicates primary endothelial cells. EK indicates endothelial cells, which can grow in reduced serum due to KSHV infection. ET indicates KSHV infected endothelial cells, which can form tumors in nude mice, such as E1 and L1 TIVE cells. KS indicates Kaposi sarcoma, which is the only cell type to experience notable levels of lytic reactivation with KSHV infection.

The mir-221 pre-miRNA emerged as a biomarker for the transition from immortalized to tumorigenic endothelial cells independent of KSHV infection status (Figure 4A). Mir-222 was co-regulated with mir-221, but did not change as dramatically (data not shown). Since mir-221/222 exhibit tumor suppressor activity in endothelial and other cancer models [55],[56],[65],[66], this suggests that the down-regulation of the mir-221 biomarker is of biological significance.

The mir-15 pre-miRNA is an example for miRNAs that exhibit the opposite pattern of transcription as mir-221. Therefore it did contribute additional information that would have improved tumor classification. It was high in tumorigenic KSHV-infected endothelial calls, KS and PEL (data not shown). There was one significant difference between mir-15 and mir-221 expression: the KSHV-negative SLK cells transcribed significantly lower levels of mir-15. In a separate analysis of only the endothelial/KS sample and excluding SLK cells (data not shown), mir-15 levels correlated closely with KSHV latent mRNA and miRNA transcription and can thus be considered KSHV –regulated.

The mir-140 pre-miRNA levels correlated linearly with tumor status. It was present at appreciable levels only in the xenograft tumors and KS biopsies, but not KSHV-infected cells grown in culture (Figure 3B, class ETM, KS). Pre-mir-140 levels did not distinguish tonsil and PEL, since 50% of PEL lines as well as all KSHV-negative lymphoma lines had only very low levels of mir-140. Hence, the utility of mir-140 as a biomarker is limited to the endothelial lineage, but not lymphatic lineage cancers.

The mir-24-2 pre-miRNA levels were strikingly elevated only in KS biopsies, not E1 xenograft tumors or PEL (Figure 3C). It therefore serves as a KS-specific biomarker and not as a marker for KSHV-associated transformation. This may have utility for clinical diagnosis, but more importantly it represents at least one molecular difference between clinical KS lesions and all available tissue culture models. In other words, any of the KS-specific mir-24-2 dependent reprogramming of target mRNA and protein levels is not captured in our current, laboratory-based understanding of KS and KSHV biology.

To establish the utility of these four biomarkers for endothelial cell tumorigenesis, we calculated cumulative density distributions (cdf) (Figure 3E–G) and a decision tree (Figure 3D). Pre-mir-221 and pre-mir-24-2 showed steep cdfs, which allowed for binary classification into positive and negative classes. Pre-mir-140 (Figure 3F) showed an almost linear cdf consistent with gradual changes among multiple sample classes. This is reflected in the minimal decision tree (Figure 3D) that computes cut-off values for each miRNA to yield the most parsimonious and accurate classification schema. Similar decision trees could be derived using other representative miRNAs from each of the clusters identified in Figure 2. We also built decision trees based on just viral pre-miRNA levels (data not shown). These were comparable to ANOVA for individual pre-miRNAs, since KSHV genome copy number (Figure S3), latent RNA levels and latent pre-miRNA levels were all correlated (they clustered together by unsupervised clustering (Figure 1, 2) and increased progressively with increasing tumorigencity.

In sum, supervised classification established (i) the presence of molecularly distinct, progressive steps of endothelial cell transformation and (ii) a set of biomarkers that distinguishes between these steps.

## Discussion

### Pre-miRNA profiling

Mature miRNA profiling has previously been used to stratify lineage types and disease progression stages. Pre-miRNA profiling has also been used successfully to stratify human tumors [27],[28],[29]. We previously profiled pre- and mature miRNAs for PEL [26] in order to establish a PEL cancer signature, and found that pre-miRNA profiling offered technical advantages as well as provided additional, non-redundant information to mature miRNA-based PEL classification. Here, using 9 primary patient biopsies and validated pre-clinical cell culture models, we have ascertained the first pre-miRNA profile of KS.

At the genomic level, we found a variety of changes between different cell lines and tissue types, but no deletions or amplifications common to all KS biopsies or all KSHV-positive samples. At the pre-miRNA level, we identified groups of cellular miRNAs that define distinctive tissue types. QPCR has been shown to be an effective form of miRNA profiling. Northern blotting has limitations including low throughput and poor sensitivity. Alternative high throughput profiling methods, like microarrays, require high concentrations of target input, show poor sensitivity for rare targets, a limited linear range and the need for post-array validation by real-time QPCR. Therefore, QPCR appears to be a better method for a limited set of targets such as the ∼650 human miRNAs, and it can be applied easily on a pre-miRNA level as well.

The miRNA genes are named according to the 60–80 bp sequence of the pre-miRNA segment [67]. Each miRNA gene locus produces one pre-miRNA, which in turn can produce one or two mature miRNAs depending on whether both strands of the mature product are inserted into the functional RISC complex. While all miRNA genes, and therefore all pre-miRNAs, are made of unique sequence, different pre-miRNAs can be processed to yield an identical mature 22-nucleotide miRNA. For instance, there are 3 different let-7a genes: let7-a-1, let-7a-2 and let-7a-3, each located on a different chromosome (9, 13, and 22, respectively) and subject to different regulatory controls. Pre-miRNA profiling but not mature miRNA profiling distinguishes between these transcripts.

How well do pre-miRNA levels correlate with mature miRNA levels? This seemingly simple question has a non-trivial answer. (i) We and others have shown that pre-miRNA levels generally correlate with mature miRNA levels [26],[28],[29],[68],[69],[70], but we also found that some pre-miRNAs were present in a slightly different pattern of expression from the mature miRNAs. The obvious example, are the aforementioned pre-miRNA paralogs, which encode the same mature miRNA, but reside on different genomic locations. Furthermore, there are well-documented instances, where SNPs affect Dicer processing [71],[72]. These exceptions are informative in their own right and only simultaneous quantification of pre- and mature miRNA levels can identify these. In the present case mature mir-221 levels were also downregulated in KS and PEL compared to non-tumorigenic controls, but for the two others (mir-140, mir-24-2) we could not establish a statistically significant pattern based on mature miRNA levels (O'Hara et al., in press). (ii) The two assays (mature miRNA and pre-miRNA) measure two different events and thus provide non-redundant information. The pre-miRNA pool represents an intermediate step and thus responds without delay to changes in cellular transcription. Pre-miRNAs are co-transcriptionally processed [73],[74]. They have a short half-life, much like mRNAs, and thus provide a sensitive read-out for the purpose of tumor profiling. By contrast, mature miRNAs are part of the relatively stable RISC complex and thus provide a time-delayed read-out of the state of the cell. (iii) The two assays (mature miRNA and pre-miRNA) have different performance characteristics. Unfortunately, these are different for each miRNA (data not shown). Even if relative levels of pre- and mature miRNAs correlate, the different assay formats for pre- and mature miRNAs have different sensitivities, different response characteristics and a different lower limit of detection (much of which is dependent on the miRNA-specific primer sequences) and thus they have a varying ability to distinguish between the presence and absence of a miRNA sequence.

### Pre-miRNA profiling defines progressive stages of endothelial cell transformation

In the case of KS and its related cell culture and animal models, each class in our collection (E, EK, ET, ETM, KS, PEL) exhibited a distinctive cellular miRNA profile (Figure 4). Even though we found some differences in the transcription pattern between individual KSHV miRNAs (unpublished), the KSHV miRNA levels as a group correlated with an increasing tumor-forming capability of infected cells (p≤10^−10^ by ANOVA of linear model). They were present in KSHV-infected HUVEC clones, high in E1/L1 cells in culture, higher in E1 mouse tumors and KS lesions and highest in PEL. Of note, the non-tumorigenic EC clones were made with JSC-1 derived KSHV [75], whereas TIVE E1/L1 clones were made from BCBL-1 derived KSHV [40], which may yield to a difference in miRNA regulation. KSHV gene copy number also increased with increasing tumorforming ability in this set of samples. At present we cannot discern whether high KSHV pre-miRNA levels are a driver for or a consequence of increased gene copy number. There are also sequence differences between other KSHV isolates that may contribute to variability among the individual samples [76]. Sequence variation is more pronounced for pre-miRNAs because of length (70 vs 22 nt) and less selective pressure for non-essential positions.

Our data support a stepwise progression towards KS based on cellular pre-miRNA patterns alone (Figure 3D) or after integration of the KSHV miRNA data (Figure 2). This model is exemplified in Figure 4. Initially, normal endothelial cells (E) are infected with KSHV to yield stage EK. Both the uninfected and infected endothelial cells share a common endothelial lineage pre-miRNA signature (Figure 4, group I). In addition, all KSHV-infected cells express low levels of KSHV miRNAs as well as a distinct group of cellular miRNAs (Figure 4, group IV). These are able to grow in reduced serum, indicating that KSHV is a transforming virus. However, these cell lines do not form tumors in mice and are therefore not oncogenic. The viral life cycle in these cells remains tightly latent. The E1 and L1 TIVE cells are infected cells that have undergone a second transformation event. As a result cells progress to the ET stage. These cells are capable of forming tumors in mice and express intermediate levels of KSHV miRNAs and a distinctive group of pre-miRNAs (Figure 4, group II). While these cells are highly transformed, similar to KS, the life cycle of the virus is still tightly latent. We were able, for the first time, to also profile primary KS biopsies. KS lesions exhibited the highest levels of KSHV miRNAs, as compared to other infected endothelial cell samples. PEL exhibited still higher levels due to a higher genome copy number. In addition, unsupervised clustering identified a group of pre-miRNAs that are highly upregulated only in primary KS lesions (Figure 4, group III). Unlike cell culture models, which are tightly latent, KS lesions are known to undergo spontaneous lytic reactivation to varying degrees [77]. In sum, each sample class profiled had a unique set of highly transcribed cellular pre-miRNAs, independent of the presence of virus, and a second set of pre-miRNAs that were dependent on the presence of KSHV.

There exists an important distinction between tumorigenicity with is a phenotype of cell culture models and tumor take, which is a phenotype of primary tumor explants. In experimental transformation models such as NIH3T3 cells tumorgenicity in immune deficient mice is conferred by the adding one or two single oncogenes. In tumor explant models tumor take is defined as how many mice will form transplantable tumors after injection of a given dose of primary tumor cells. Tumor take is highly variable among cancer types and even individuals. It does necessarily correlate with clinical aggressiveness and does not easily correlate with a single gene. KS and EBV+ nasopharyngeal carcinoma are examples of highly aggressive, angiogenic tumors, which almost never yield stable cell lines in culture or transplantable xenografts in nude mice.

### Individual miRNAs emerge as novel tumor-stage specific biomarkers with important biological functions

Mir-221 is a tumor suppressor for endothelial cell lineage cancers independent of KSHV infection. We found the highest levels of pre-mir-221 in uninfected and KSHV latently infected tert-HUVEC and tert-HMVEC cell lines. This corroborates prior reports of high mir-221 levels in endothelial cell lines [66],[78],[79]. High levels of mir-221 exert anti-angiogenic effects in HUVEC cells, resulting in inhibited tube formation, migration and wound healing [66]. This anti-angiogenic effect correlated with downregulated expression of the mir-221 target protein c-kit [66]. In Dicer siRNA-transfected cells, mir-221 expression has also been shown to indirectly downregulate expression of endothelial nitric oxide synthase (eNOS) [78]. Nitric oxide is a key regulator of endothelial cell growth, migration, vascular remodeling and angiogenesis. The picture is more complicated, though, since depletion of mir-221 in HUVEC cells causes secondary changes in other miRNAs [66],[80]; these included many that are predicted to also target c-kit. C-kit expression was also reduced by mir-221 in hematopoietic progenitor cells. In this system, mir-221 also inhibited proliferation [81]. Additional targets for mir-221 include CDKN1B/p27 and CDKN1C/p57, which are cell cycle regulators [56],[59],[65],[82]. Disregulation of mir-221 has been found in melanomas due to silencing of the promyelocytic leukemia zinc finger (PLZF) transcription factor [83]. In summary, mir-221 seems to possess endothelial cell lineage-specific differentiation functions as well as general tumor/proliferation suppressor functions.

Pre-mir-34a and c were found at detectable levels in endothelial cells, PEL and KS, as these tumors retain wild-type p53 (Figure 1). The miR-34 promoter is p53-responsive [61],[64],[84],[85],[86],[87]. Of all three p53-responsive miRNAs, mir-34a appears to be the most responsive in terms of fold change [61]. High levels of miR-34 are consistent with the biology of PEL and KS, which are unusual among human cancers because they almost universally retain fully functional, wild type p53 [88],[89]. Three different miR-34 genes are present in the human genome. Mir-34a is located within the second exon of a non-coding gene, which contains a predicted p53-binding site. Genes mir-34b and mir-34c are located within a single non-coding precursor with a transcriptional start site adjacent to a predicted p53-binding site [86]. All three genes produce mature miRNAs with an identical seed sequence. It will be interesting to determine whether mir-34a, similar to p53-responsive mRNAs [88], can be even further induced upon chemotherapy in PEL and KS.

Pre-mir-140 levels were tightly correlated with KSHV latent mRNA (LANA) and latent miRNA levels in KS and KS tumor models. Currently, little is known regarding mir-140 expression profiles or possible mRNA targets [90]. TargetScan miRNA target software indicates a number of possible targets for mir-140, including E2F3, a member of the E2F family of transcription factors essential for cell cycle regulation. This prediction, however, awaits experimental verification.

Pre-mir-24 emerged as a highly specific KS biomarker compared to all other pre-miRNAs in our array. Mir-24 has been shown to be important in cell-cycle regulation, cell growth and differentiation in a variety of cell types [91],[92]. However, these as well as functional studies on mir-24 and its targets are still in the early stages. Tantalizing data predict p16 and dehydrofolate reductase (DHFR) as mir-24 targets among others [93],[94],[95],[96],[97].

In summary, the first pre-miRNA profiling of primary KS tumor biopsies and the subsequent comparison to well-studied culture and mouse xenograft models of KS yielded a progression model for endothelial lineage cancer and KS (Figure 4) akin to the now classical model for colorectal cancer progression [98]. We hope that this will benefit basic and translational studies of KS, which remains the most frequent cancer in people living with HIV/AIDS today. We also identified specific KSHV and KS-associated pre-miRNAs, foremost among them mir-221, mir-140, mir-15a and mir-24. Based upon their strength of association with specific stages of endothelial cell tumor progression, we speculate that these are also functionally involved in KS tumorigenesis.

## Materials and Methods

### Cell culture and clinical biopsies

Cells were grown in continuous culture on a 3T3-like schedule, i.e. passaged at subconfluency, and RNA collected in log phase, typically 24–48 hrs after reseeding. All B and T cells were cultured in RPMI containing 25mM HEPES, 10% fetal bovine serum (AP2, AP3, AP5 in 20%), 0.05 mM 2-mercaptoethanol, 1 mM sodium pyruvate, 2 mM L-glutamine, 0.05 ug penicillin/mL, and 20 U streptomycin/mL at 37° and in 5% CO_2_. TIVE cells were cultured in DMEM containing 10% fetal bovine serum, 2 mM L-glutamine, 0.05 ug penicillin/mL, and 20 U streptomycin/mL at 37° and in 5% CO_2_. HUVEC-hTERT cells were cultured in EGM-2 containing 10% fetal bovine serum, hydrocortisone, hRGF, VEGF, R3-IGF, Ascorbic acid, hEGF, GA-1000 and heparin at 37° and in 5% CO_2_. HUVEC+KSHV cells were cultured in the same media also containing 0.5pM/ul puromycin to maintain selection. De-identified frozen tonsil and melanoma tissue biopsies were obtained from the cooperative human tissue network (CHTN). KS frozen tissue biopsies were obtained after informed consent at University of Miami, Prof. Edgard Santos University Hospital, Salvador, Brazil and Beth Israel Deaconess Medical Center. All cell lines and references are described in Table S1.

### DNA and RNA isolation

DNA was isolated from cell lines and samples using the Wizard SV Genomic kit (Promega, Madison, WI). Total RNA was isolated using Triazol (Sigma-Aldrich, St Louis, MO) as previously described [78]. Total RNA was quantitated on a Nanodrop and equal amounts of RNA were subjected to DNase I treatment (Ambion, Austin, TX). RNA was reversed transcribed using the cDNA Archive Kit (Applied Biosystems, Foster City, CA), with the addition of RNase Inhibitor and, in pre-miRNA screening, the additions of T4 gene protein 32. RNA integrity was evaluated using a 2100 Bioanalyzer Series C (Agilent, Santa Clara, CA). Total RNA was measured using the RNA 6000 Series II Nano kit and small RNA was measured using the Small RNA kit, according to the manufacturers recommendations. All Chips were analyzed using 2100 Expert software version B.02.04. The average RNA integrity value for total RNA among all samples profiled was 8.00±2.60.

### Real-time QPCR

For DNA and pre-miRNA expression profiling, two 96-well plates containing 372 different primers were used. These primers represent 168 cellular and 12 viral pre-miRNA targets, as well as 6 cellular and viral control miRNA targets. All primers conform to universal real-time PCR conditions with a predicted Tm of 60 and 100-bp or smaller amplicon length. Real-Time QPCR was conducted under universal cycling conditions of 40 cycles with SYBR Green as the method of detection following our previously validated methods. A 36ul reaction mix was made using a CAS-1200 robot that uses filtered carbon-graphite pipette tips (Tecan Inc., Durham, NC) for liquid level sensing, allowing for a pipetting accuracy of 0.1ul. The reaction mix was then distributed in triplicate into a 384-well plate using a Matrix repeat pipettor (Thermo Inc.). The final primer concentration was 250nM in total of the 9ul reaction volume. Because pre-miRNA-specific primers also detect the corresponding gene, these primers were used for DNA gene profiling as well. For DNA QPCR, each reaction contained 1.67ng DNA/ul. For pre-miRNA QPCR, 40ul of the 100ul RT reaction was used for each 384 well plate, yielding a final amount of 0.1ul cDNA per each 9ul reaction. Real-time QPCR primers against 93 mature miRNAs and 1 cellular mRNA were used according to the manufacturers protocol (Applied Biosystems Inc.). The combined pipetting and instrument error for all of the QPCR reactions was less than 6% (data not shown). All reactions were done in technical triplicates. QPCR was performed on a 384 well LC480 (Roche Inc.) platform.

### Statistical analysis

Data were collected in triplicate for each RT reaction. Since averaging these replicates would mask individual reaction failures, we clustered all replicates individually after masking outliers. Each array of 160 primers contained four separate reactions for U6 yielding six CT_U6_. We calculated the mean and median of these four reactions to yield ˆCT_U6_. The maximal difference between mean and median was 0.30 CT units. All other CT data were normalized to ˆCT_U6_. This yielded dCT for each primer/sample combination. The dCT were normalized to median for each array and subjected to unsupervised clustering using a Correlation metric [51] and the program Arrayminer™.

Our exploratory cluster analysis included all primers and all samples. However, for statistical analysis we excluded primers, which yielded CT <38 for the non-template and reverse transcriptase negative controls. We also excluded primers, which did not yield a signal (CT<38) in at least one of the samples as uninformative. We used QQ plots for each sample and Kolgomoroff-Smirnoff statistics to test for normal distribution across arrays (data not shown).

For miRNA gene copy number analysis, data were collected in triplicate (or duplicate) for each sample. Since averaging these replicates would mask individual reaction failures, we clustered all replicates individually after masking outliers. Each set of 160 primer pairs contained four separate reactions for U6 yielding four CT_U6_. We calculated the mean of these four reactions to yield ˆCT_U6_. In only two samples did the median deviate significantly from the mean based on an analysis of residuals (data not shown). This suggested individual reaction or pipetting failures. We imputed modified ˆCT_U6_ for those singular cases based on the following rule: if <50% of replicates differed by >1 CT from the mean of the remainder, they were replaced by the mean of the remaining data points. After imputation, the mean ˆCT_U6_ across all samples and all technical replicates (n = 348) was 20.94±1.97 with a median of 21.26. For individual quadruplicate CT_U6_ measurements, the SDs ranged from 0.03 to 1.00 CT. 71.26% of SDs for technical replicates were ≤0.32 CTs. Hence, this array identified 2-fold changes in copy number. All other CT data were normalized to ˆCT_U6_. This yielded dCT for each primer/sample combination. The dCT were subjected to unsupervised clustering using an Euklidian metric and visualized on a log2 scale [51].

For supervised comparisons between two classes, we used the Welch-modified t-test as implemented in the R statistics program [99]. This yielded unadjusted, univariate p values for each individual miRNA gene. This particular variant of the t-test allows for unequal variances between the two classes. An analysis of variances showed that most miRNA genes had identical variances between the KSHV-infected (n = 53) and normal tissue (n = 18) data sets. We used q-value computation [100], to assess the false discovery rate. The statistical methods for supervised comparisons between two classes of pre-miRNAs were as described above for miRNA gene loci. We only report the minimal set of miRNA genes for which we do not expect any false positives. The Bonferroni-adjusted p-value was ≤0.05 for each of the hits. Decision trees were computed as implemented in R [99] using 10 fold cross-validation.

### Quality control

**Figure 5:**
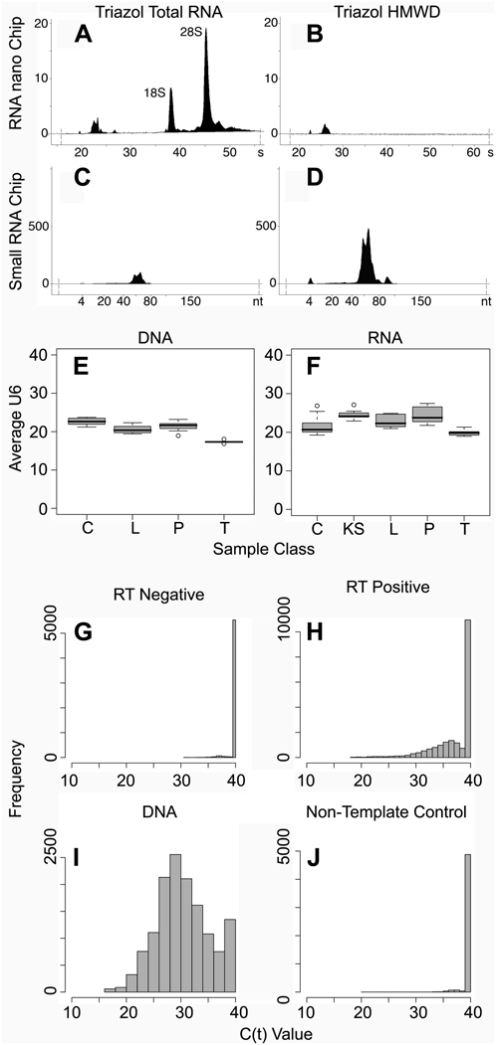
QPCR quality control. Agilent Bioanalyzer™ RNA chip results are shown for two common extraction methods of RNA. The BC2 cell line was used. (A) The RNA nanochip on Triazol™ extracted total RNA has a calculated concentration of 237.5 ng/ul, using peak comparison, and a RNA integrity value of 9.7, while (B) the high molecular weight deleted sample (HMWD) is depleted for the rRNA and tRNA necessary for these calculations. (C) The small RNA chip on the total RNA sample has a concentration of 20.5 pg/µl as measured by area between 20–40 nucleotide while (D) the HMWD depleted sample has only 10.5 pg/µl in the same region. Average U6 values of all samples for the DNA (E) and RNA (F) are shown in box and whisker plots. C indicates endothelial cell culture models, both infected and uninfected, KS indicates primary KS biopsies, L indicates B and T cell lymphoma cell lines, P indicates PEL, and T indicates Tonsil Tissue. Outliers are shown in open dots. Histograms of individual QPCR reactions are shown for all reaction types, with frequency shown on the y-axis and CT value shown on the x-axis. A CT value ≤38.00 is considered positive. (G) For all RT negative reactions (n = 5,920), 4.83% are positive. (H) For all RT positive reactions (n = 20,000), 41.50% are positive. (I) For all DNA (n = 13,920), reactions 90.28% are positive. (J) For all non-template water control reactions (n = 5,120), 3.96% are positive.

To monitor RNA integrity and yield, we used the Agilent bioanalyzer (Figure 5A–D). The Agilent bioanalyzer provides two chips with different size resolution. The small RNA chip resolves RNA species from 4–150 nt (Figure 5C,D), the RNA nano chip RNA species from 25–6000 nt (Figure 5A,B). It allows size determination as well as quantitation. We compared two RNA preparations: total RNA isolated with Triazol and total RNA isolated with Triazol followed by high molecular weight depletion (HMWD). The total RNA isolation with Triazol™ retained the highest concentration of miRNAs (20.5 pg/µl) while preserving overall RNA integrity as measured by RIN value (9.7), which is a proprietary estimate based on ratio of 28S to 18S peak (Figure 5A). Subsequent HMWD as required for other mature miRNA profiling approaches (e.g. [101]) depleted mRNA and rRNA, as expected. It did not change the relative abundance of miRNA (∼22nt) to pre-miRNA ∼70 nt), but decreased overall small RNA yield by half (10.5 pg/µl). Hence, we used total RNA for all further studies.

We used real-time QPCR to determine individual pre-miRNA levels as per our published procedures [26]. Individual miRNA CT readings were normalized to CT of U6 rRNA (dCT_U6_) to account for variation in sample input RNA or DNA. However, we generally obtained more consistent results if we used very similar amounts of RNA (as determined by nanodrop™ based quantitation) for the reverse transcriptase (RT) reaction. The reason for this pre-RT normalization step is that the RT reaction, too, has a linear range just as the real-time QPCR reaction, otherwise the RT reaction may be saturated or, in case of diluted samples, of lower than expected RT efficiency. Figure 5E shows average raw CT values for U6 for each DNA sample, aggregated by sample class. Figure 3F shows average CT values for U6 for each post RT cDNA sample, aggregated by sample class. Again, variation is minimal except for two outliers (HMVEC and KS 101), for which we had only small amounts of RNA available. Though even those two samples had U6 CT values of ≤27 cycles. Since we ran a 40 cycle QPCR reaction, this gave us an assay range of 2^(27-40 = 13)^ = 0 to 8192 fold above the level of detection.

All experimental samples were run in triplicate for RT-positive and DNA reactions. Pooled samples were run in triplicate for RT-negative control reactions. Non-template control (NTC) reactions were run to assure that the primers were free of contamination and did not yield non-specific products or primer dimers at a significant rate. A reaction was considered positive if the corresponding CT was greater than 38.00 cycles. To remove primers with substantial capacity for primer dimer formation, any primer pair with mean CT <38.00 in the RT- or NTC reactions was omitted from further analysis. This removed 8 cellular pre-miRNA and 1 viral miRNA primer pairs (KSHV-mir-k12-10a) from our original set [26]. Conversely, any primer pair that failed to efficiently amplify the corresponding DNA target (mean CT_DNA_ >38.00) was also omitted from further analysis. This filtering removed 15 cellular pre-miRNA primer pairs, 1 viral miRNA primer (KSHV-mir-k12-3). It also removed all no primer controls from the data set. In all, the final array included 160 primer pairs, representing 145 cellular miRNAs, 6 KSHV miRNAs, 3 EBV miRNAs, 2 KSHV mRNAs and 4 cellular rRNAs (U6). These were run in parallel for each sample. The distribution of all data used in the analysis was as follows: for the NTC (Figure 5J), 203 of 5120 reactions (3.96%) of the reactions had a CT<38. These were randomly distributed across all samples and all primers. For the RT- reactions (Figure 5G), 286 of 5920 (4.83%) of the reactions had a CT<38. Pair-wise comparison of the RT- and NTC results indicated perfect overlap between the positive samples indicating these were incidences of shared positivity and should therefore only be counted once (data not shown). Therefore the false positive rate was 4.83%. In the DNA samples (Figure 5I), 12,567 of 13,920 (90.28%) of the reactions had CT<38. Given that less than 5% of the total number of reactions represent viral targets, the presence of which is variable from sample to sample, the remaining negative values most likely indicate deletions of miRNAs in certain samples. The distribution was unimodal and followed a normal distribution (data not shown). Finally, the RT-positive (Figure 5H) set contained 8,299 of 20,000 (41.50%) positive reactions (CT <38) in total. These are less than half the positive reactions recorded for the corresponding DNA results. This means that although we could detect 86% of all miRNA genes (DNA) in all samples, only 45.54% were actively transcribed in our set of samples. This means that half of all miRNAs in our array were transcribed in the endothelial cell lineage. This is consistent with the known tissue specificity of miRNAs and underscores their value as differentially expressed biomarkers. This is likely due to a combination of factors, including tissue type and developmental stage. For instance, we would not expect liver or brain-specific miRNAs to be present in any of our samples.

## Supporting Information

Figure S1 Minimal influence of proliferation phase on pre-miRNA levels in E1 and L1 cells. (A) Clustering analysis indicates few miRNAs changed in expression pattern during growth phases. (B) Principal component analysis of clustering indicates non-overlapping clusters.
(1.57 MB TIF)

Figure S2 Pair-wise comparisons of dCT values for pre-miRNAs. (A) HUVEC against HMVEC, (B) KSHV-infected HUVEC against EC, (C) E1/L1 against KSHV-infected HUVEC, (D) E1/L1 cells in culture against E1 tumors, (E) KS biopsies against E1 tumors, (F) KS tumors against E1/L1 cell lines. Plotted are median dCT_U6_ values.
(2.66 MB TIF)

Figure S3 Relative KSHV genome copy number (2^dCTU6^) based on real-time QPCR for the LANA orf. The classes are (E) uninfected endothelial cells, (EK) KSHV infected endothelial cells, (ET) KSHV-infected endothelial cells, which can form tumors in nude mice (E1 and L1 TIVE), (P) Pel lines, (TN) tonsil. Shown also is the fold difference in relative copy number between KSHV-positive classes. Neither E nor RN yielded a detecable signal (1 copy per <100,000 cell equivalents).
(2.26 MB TIF)

Figure S4 Pre-miRNA profiling throughout the cell cycle indicates few miRNAs are changed. (A) Growth curves for the E1 (red dots) and L1 (blue triangles) cell lines. Number of cells per T75 flask is shown on vertical axis and time in hours since seeding on the horizontal axis. Logarithmic indicates the time points that were averaged to yield logarithmic phase pre-miRNA levels and Stationary indicates time points that were averaged to yield stationary phase pre-miRNA levels. (B–E) Pair-wise comparisons of average log-pre-miRNA levels (dCT_U6_) of proliferation phases and cell lines. (Only data points with SD ≤3 were included in the comparison).
(1.94 MB TIF)

Table S1 Indicated are the samples profiled, viral status and original cell line reference paper or sample source.
(0.08 MB DOC)

## References

[1] El Amari EB, Toutous-Trellu L, Gayet-Ageron A, Baumann M, Cathomas G (2008). Predicting the evolution of Kaposi sarcoma, in the highly active antiretroviral therapy era.. Aids.

[2] Franceschi S, Maso LD, Rickenbach M, Polesel J, Hirschel B (2008). Kaposi sarcoma incidence in the Swiss HIV Cohort Study before and after highly active antiretroviral therapy.. Br J Cancer.

[3] Krown SE, Lee JY, Dittmer DP (2008). More on HIV-associated Kaposi's sarcoma.. N Engl J Med.

[4] Ghosh K, Thodeti CK, Dudley AC, Mammoto A, Klagsbrun M (2008). Tumor-derived endothelial cells exhibit aberrant Rho-mediated mechanosensing and abnormal angiogenesis in vitro.. Proc Natl Acad Sci U S A.

[5] Qiu W, Hu M, Sridhar A, Opeskin K, Fox S (2008). No evidence of clonal somatic genetic alterations in cancer-associated fibroblasts from human breast and ovarian carcinomas.. Nat Genet.

[6] Hu M, Yao J, Cai L, Bachman KE, van den Brule F (2005). Distinct epigenetic changes in the stromal cells of breast cancers.. Nat Genet.

[7] Martin JN, Ganem DE, Osmond DH, Page-Shafer KA, Macrae D (1998). Sexual transmission and the natural history of human herpesvirus 8 infection.. N Engl J Med.

[8] Gao S-J, Kingsley L, Hoover DR, Spira TJ, Rinaldo CR (1996). Seroconversion to antibodies against Kaposi's sarcoma-associated herpesvirus-related latent nuclear antigens before the development of Kaposi's sarcoma.. New England Journal of Medicine.

[9] Chang Y, Cesarman E, Pessin MS, Lee F, Culpepper J (1994). Identification of herpesvirus-like DNA sequences in AIDS-associated Kaposi's sarcoma [see comments].. Science.

[10] Dittmer D, Lagunoff M, Renne R, Staskus K, Haase A (1998). A cluster of latently expressed genes in Kaposi's sarcoma-associated herpesvirus.. J Virol.

[11] Dupin N, Fisher C, Kellam P, Ariad S, Tulliez M (1999). Distribution of human herpesvirus-8 latently infected cells in Kaposi's sarcoma, multicentric Castleman's disease, and primary effusion lymphoma.. Proceedings of the National Academy of Science USA.

[12] Cesarman E, Chang Y, Moore PS, Said JW, Knowles DM (1995). Kaposi's sarcoma-associated herpesvirus-like DNA sequences in AIDS- related body-cavity-based lymphomas [see comments].. N Engl J Med.

[13] Soulier J, Grollet L, Oksenhendler E, Cacoub P, Cazals-Hatem D (1995). Kaposi's sarcoma-associated herpesvirus-like DNA sequences in multicentric Castleman's disease [see comments].. Blood.

[14] Calin GA, Croce CM (2006). MicroRNA signatures in human cancers.. Nat Rev Cancer.

[15] Griffiths-Jones S, Saini HK, van Dongen S, Enright AJ (2008). miRBase: tools for microRNA genomics.. Nucleic Acids Res.

[16] Pfeffer S, Sewer A, Lagos-Quintana M, Sheridan R, Sander C (2005). Identification of microRNAs of the herpesvirus family.. Nat Methods.

[17] Samols MA, Hu J, Skalsky RL, Renne R (2005). Cloning and identification of a microRNA cluster within the latency-associated region of Kaposi's sarcoma-associated herpesvirus.. J Virol.

[18] Grundhoff A, Sullivan CS, Ganem D (2006). A combined computational and microarray-based approach identifies novel microRNAs encoded by human gamma-herpesviruses.. Rna.

[19] Cai X, Lu S, Zhang Z, Gonzalez CM, Damania B (2005). Kaposi's sarcoma-associated herpesvirus expresses an array of viral microRNAs in latently infected cells.. Proc Natl Acad Sci U S A.

[20] Skalsky RL, Samols MA, Plaisance KB, Boss IW, Riva A (2007). Kaposi's sarcoma-associated herpesvirus encodes an ortholog of miR-155.. J Virol.

[21] Gottwein E, Mukherjee N, Sachse C, Frenzel C, Majoros WH (2007). A viral microRNA functions as an orthologue of cellular miR-155.. Nature.

[22] Samols MA, Skalsky RL, Maldonado AM, Riva A, Lopez MC (2007). Identification of cellular genes targeted by KSHV-encoded microRNAs.. PLoS Pathog.

[23] Landgraf P, Rusu M, Sheridan R, Sewer A, Iovino N (2007). A mammalian microRNA expression atlas based on small RNA library sequencing.. Cell.

[24] Volinia S, Calin GA, Liu CG, Ambs S, Cimmino A (2006). A microRNA expression signature of human solid tumors defines cancer gene targets.. Proc Natl Acad Sci U S A.

[25] Yanaihara N, Caplen N, Bowman E, Seike M, Kumamoto K (2006). Unique microRNA molecular profiles in lung cancer diagnosis and prognosis.. Cancer Cell.

[26] O'Hara AJ, Vahrson W, Dittmer DP (2008). Gene alteration and precursor and mature microRNA transcription changes contribute to the miRNA signature of primary effusion lymphoma.. Blood.

[27] Lee EJ, Gusev Y, Jiang J, Nuovo GJ, Lerner MR (2007). Expression profiling identifies microRNA signature in pancreatic cancer.. Int J Cancer.

[28] Jiang J, Lee EJ, Gusev Y, Schmittgen TD (2005). Real-time expression profiling of microRNA precursors in human cancer cell lines.. Nucleic Acids Res.

[29] Lee EJ, Baek M, Gusev Y, Brackett DJ, Nuovo GJ (2008). Systematic evaluation of microRNA processing patterns in tissues, cell lines, and tumors.. RNA.

[30] Flore O, Rafii S, Ely S, O'Leary JJ, Hyjek EM (1998). Transformation of primary human endothelial cells by Kaposi's sarcoma- associated herpesvirus.. Nature.

[31] Ciufo DM, Cannon JS, Poole LJ, Wu FY, Murray P (2001). Spindle cell conversion by Kaposi's sarcoma-associated herpesvirus: formation of colonies and plaques with mixed lytic and latent gene expression in infected primary dermal microvascular endothelial cell cultures.. J Virol.

[32] Grundhoff A, Ganem D (2004). Inefficient establishment of KSHV latency suggests an additional role for continued lytic replication in Kaposi sarcoma pathogenesis.. J Clin Invest.

[33] Lagunoff M, Bechtel J, Venetsanakos E, Roy AM, Abbey N (2002). De novo infection and serial transmission of Kaposi's sarcoma-associated herpesvirus in cultured endothelial cells.. J Virol.

[34] Grossmann C, Podgrabinska S, Skobe M, Ganem D (2006). Activation of NF-kappaB by the latent vFLIP gene of Kaposi's sarcoma-associated herpesvirus is required for the spindle shape of virus-infected endothelial cells and contributes to their proinflammatory phenotype.. J Virol.

[35] Carroll PA, Brazeau E, Lagunoff M (2004). Kaposi's sarcoma-associated herpesvirus infection of blood endothelial cells induces lymphatic differentiation.. Virology.

[36] Wang L, Damania B (2008). Kaposi's sarcoma-associated herpesvirus confers a survival advantage to endothelial cells.. Cancer Res.

[37] Moses AV, Fish KN, Ruhl R, Smith PP, Strussenberg JG (1999). Long-term infection and transformation of dermal microvascular endothelial cells by human herpesvirus 8.. J Virol.

[38] Wang HW, Trotter MW, Lagos D, Bourboulia D, Henderson S (2004). Kaposi sarcoma herpesvirus-induced cellular reprogramming contributes to the lymphatic endothelial gene expression in Kaposi sarcoma.. Nat Genet.

[39] Hong YK, Foreman K, Shin JW, Hirakawa S, Curry CL (2004). Lymphatic reprogramming of blood vascular endothelium by Kaposi sarcoma-associated herpesvirus.. Nat Genet.

[40] An FQ, Folarin HM, Compitello N, Roth J, Gerson SL (2006). Long-Term-Infected Telomerase-Immortalized Endothelial Cells: a Model for Kaposi's Sarcoma-Associated Herpesvirus Latency In Vitro and In Vivo.. J Virol.

[41] Mutlu AD, Cavallin LE, Vincent L, Chiozzini C, Eroles P (2007). In Vivo-Restricted and Reversible Malignancy Induced by Human Herpesvirus-8 KSHV: A Cell and Animal Model of Virally Induced Kaposi's Sarcoma.. Cancer Cell.

[42] Herndier BG, Werner A, Arnstein P, Abbey NW, Demartis F (1994). Characterization of a human Kaposi's sarcoma cell line that induces angiogenic tumors in animals.. Aids.

[43] Nador RG, Cesarman E, Chadburn A, Dawson DB, Ansari MQ (1996). Primary effusion lymphoma: a distinct clinicopathologic entity associated with the Kaposi's sarcoma-associated herpes virus.. Blood.

[44] Fan W, Bubman D, Chadburn A, Harrington WJ, Cesarman E (2005). Distinct subsets of primary effusion lymphoma can be identified based on their cellular gene expression profile and viral association.. J Virol.

[45] Klein U, Gloghini A, Gaidano G, Chadburn A, Cesarman E (2003). Gene expression profile analysis of AIDS-related primary effusion lymphoma (PEL) suggests a plasmablastic derivation and identifies PEL-specific transcripts.. Blood.

[46] Staskus KA, Zhong W, Gebhard K, Herndier B, Wang H (1997). Kaposi's sarcoma-associated herpesvirus gene expression in endothelial (spindle) tumor cells.. J Virol.

[47] Sturzl M, Blasig C, Schreier A, Neipel F, Hohenadl C (1997). Expression of HHV-8 latency-associated T0.7 RNA in spindle cells and endothelial cells of AIDS-associated, classical and African Kaposi's sarcoma.. Int J Cancer.

[48] Boshoff C, Schulz TF, Kennedy MM, Graham AK, Fisher C (1995). Kaposi's sarcoma-associated herpesvirus infects endothelial and spindle cells.. Nat Med.

[49] Li JJ, Huang YQ, Cockerell CJ, Friedman-Kien AE (1996). Localization of human herpes-like virus type 8 in vascular endothelial cells and perivascular spindle-shaped cells of Kaposi's sarcoma lesions by in situ hybridization.. Am J Pathol.

[50] Renne R, Lagunoff M, Zhong W, Ganem D (1996). The size and conformation of Kaposi's sarcoma-associated herpesvirus (human herpesvirus 8) DNA in infected cells and virions.. J Virol.

[51] Eisen MB, Spellman PT, Brown PO, Botstein D (1998). Cluster analysis and display of genome-wide expression patterns.. Proc Natl Acad Sci U S A.

[52] Simon RM, Korn EL, McShane LM, Radmacher MD, Wright GW (2003). Design and Analysis of DNA Microarray Investigations..

[53] Troyanskaya OG, Dolinski K, Owen AB, Altman RB, Botstein D (2003). A Bayesian framework for combining heterogeneous data sources for gene function prediction (in Saccharomyces cerevisiae).. Proc Natl Acad Sci U S A.

[54] Cheadle C, Vawter MP, Freed WJ, Becker KG (2003). Analysis of microarray data using Z score transformation.. J Mol Diagn.

[55] Medina R, Zaidi SK, Liu CG, Stein JL, van Wijnen AJ (2008). MicroRNAs 221 and 222 bypass quiescence and compromise cell survival.. Cancer Res.

[56] Galardi S, Mercatelli N, Giorda E, Massalini S, Frajese GV (2007). miR-221 and miR-222 expression affects the proliferation potential of human prostate carcinoma cell lines by targeting p27Kip1.. J Biol Chem.

[57] Iyer VR, Eisen MB, Ross DT, Schuler G, Moore T (1999). The transcriptional program in the response of human fibroblasts to serum [see comments].. Science.

[58] Liu Q, Fu H, Sun F, Zhang H, Tie Y (2008). miR-16 family induces cell cycle arrest by regulating multiple cell cycle genes.. Nucleic Acids Res.

[59] Visone R, Russo L, Pallante P, De Martino I, Ferraro A (2007). MicroRNAs (miR)-221 and miR-222, both overexpressed in human thyroid papillary carcinomas, regulate p27Kip1 protein levels and cell cycle.. Endocr Relat Cancer.

[60] Vasudevan S, Steitz JA (2007). AU-rich-element-mediated upregulation of translation by FXR1 and Argonaute 2.. Cell.

[61] Tarasov V, Jung P, Verdoodt B, Lodygin D, Epanchintsev A (2007). Differential regulation of microRNAs by p53 revealed by massively parallel sequencing: miR-34a is a p53 target that induces apoptosis and G1-arrest.. Cell Cycle.

[62] Linsley PS, Schelter J, Burchard J, Kibukawa M, Martin MM (2007). Transcripts targeted by the microRNA-16 family cooperatively regulate cell cycle progression.. Mol Cell Biol.

[63] Hwang HW, Wentzel EA, Mendell JT (2007). A hexanucleotide element directs microRNA nuclear import.. Science.

[64] Chang TC, Wentzel EA, Kent OA, Ramachandran K, Mullendore M (2007). Transactivation of miR-34a by p53 broadly influences gene expression and promotes apoptosis.. Mol Cell.

[65] le Sage C, Nagel R, Egan DA, Schrier M, Mesman E (2007). Regulation of the p27(Kip1) tumor suppressor by miR-221 and miR-222 promotes cancer cell proliferation.. EMBO J.

[66] Poliseno L, Tuccoli A, Mariani L, Evangelista M, Citti L (2006). MicroRNAs modulate the angiogenic properties of HUVECs.. Blood.

[67] Griffiths-Jones S, Grocock RJ, van Dongen S, Bateman A, Enright AJ (2006). miRBase: microRNA sequences, targets and gene nomenclature.. Nucleic Acids Res.

[68] Xia T, O'Hara A, Araujo I, Barreto J, Carvalho E (2008). EBV microRNAs in primary lymphomas and targeting of CXCL-11 by ebv-mir-BHRF1-3.. Cancer Res.

[69] Schmittgen TD, Lee EJ, Jiang J, Sarkar A, Yang L (2008). Real-time PCR quantification of precursor and mature microRNA.. Methods.

[70] Schmittgen TD, Jiang J, Liu Q, Yang L (2004). A high-throughput method to monitor the expression of microRNA precursors.. Nucleic Acids Res.

[71] Gottwein E, Cai X, Cullen BR (2006). A novel assay for viral microRNA function identifies a single nucleotide polymorphism that affects Drosha processing.. J Virol.

[72] Duan R, Pak C, Jin P (2007). Single nucleotide polymorphism associated with mature miR-125a alters the processing of pri-miRNA.. Hum Mol Genet.

[73] Cai X, Hagedorn CH, Cullen BR (2004). Human microRNAs are processed from capped, polyadenylated transcripts that can also function as mRNAs.. Rna.

[74] Morlando M, Ballarino M, Gromak N, Pagano F, Bozzoni I (2008). Primary microRNA transcripts are processed co-transcriptionally.. Nat Struct Mol Biol.

[75] Vieira J, O'Hearn PM (2004). Use of the red fluorescent protein as a marker of Kaposi's sarcoma-associated herpesvirus lytic gene expression.. Virology.

[76] Marshall V, Parks T, Bagni R, Wang CD, Samols MA (2007). Conservation of Virally Encoded MicroRNAs in Kaposi Sarcoma-Associated Herpesvirus in Primary Effusion Lymphoma Cell Lines and in Patients with Kaposi Sarcoma or Multicentric Castleman Disease.. J Infect Dis.

[77] Dittmer DP (2003). Transcription profile of Kaposi's sarcoma-associated herpesvirus in primary Kaposi's sarcoma lesions as determined by real-time PCR arrays.. Cancer Res.

[78] Suarez Y, Fernandez-Hernando C, Pober JS, Sessa WC (2007). Dicer dependent microRNAs regulate gene expression and functions in human endothelial cells.. Circ Res.

[79] Kuehbacher A, Urbich C, Zeiher AM, Dimmeler S (2007). Role of Dicer and Drosha for endothelial microRNA expression and angiogenesis.. Circ Res.

[80] Tuccoli A, Poliseno L, Rainaldi G (2006). miRNAs regulate miRNAs: coordinated transcriptional and post-transcriptional regulation.. Cell Cycle.

[81] Felli N, Fontana L, Pelosi E, Botta R, Bonci D (2005). MicroRNAs 221 and 222 inhibit normal erythropoiesis and erythroleukemic cell growth via kit receptor down-modulation.. Proc Natl Acad Sci U S A.

[82] Fornari F, Gramantieri L, Ferracin M, Veronese A, Sabbioni S (2008). MiR-221 controls CDKN1C/p57 and CDKN1B/p27 expression in human hepatocellular carcinoma.. Oncogene.

[83] Felicetti F, Errico MC, Bottero L, Segnalini P, Stoppacciaro A (2008). The promyelocytic leukemia zinc finger-microRNA-221/-222 pathway controls melanoma progression through multiple oncogenic mechanisms.. Cancer Res.

[84] Welch C, Chen Y, Stallings RL (2007). MicroRNA-34a functions as a potential tumor suppressor by inducing apoptosis in neuroblastoma cells.. Oncogene.

[85] Raver-Shapira N, Marciano E, Meiri E, Spector Y, Rosenfeld N (2007). Transcriptional activation of miR-34a contributes to p53-mediated apoptosis.. Mol Cell.

[86] He L, He X, Lim LP, de Stanchina E, Xuan Z (2007). A microRNA component of the p53 tumour suppressor network.. Nature.

[87] Corney DC, Flesken-Nikitin A, Godwin AK, Wang W, Nikitin AY (2007). MicroRNA-34b and MicroRNA-34c are targets of p53 and cooperate in control of cell proliferation and adhesion-independent growth.. Cancer Res.

[88] Petre CE, Sin SH, Dittmer DP (2007). Functional p53 signaling in Kaposi's sarcoma-associated herpesvirus lymphomas: implications for therapy.. J Virol.

[89] Katano H, Sato Y, Sata T (2001). Expression of p53 and human herpesvirus-8 (HHV-8)-encoded latency-associated nuclear antigen with inhibition of apoptosis in HHV-8-associated malignancies.. Cancer.

[90] Tuddenham L, Wheeler G, Ntounia-Fousara S, Waters J, Hajihosseini MK (2006). The cartilage specific microRNA-140 targets histone deacetylase 4 in mouse cells.. FEBS Lett.

[91] Wang X, Tang S, Le SY, Lu R, Rader JS (2008). Aberrant expression of oncogenic and tumor-suppressive microRNAs in cervical cancer is required for cancer cell growth.. PLoS ONE.

[92] Merkerova M, Belickova M, Bruchova H (2008). Differential expression of microRNAs in hematopoietic cell lineages.. Eur J Haematol.

[93] Lal A, Kim HH, Abdelmohsen K, Kuwano Y, Pullmann R (2008). p16(INK4a) translation suppressed by miR-24.. PLoS ONE.

[94] Sun Q, Zhang Y, Yang G, Chen X, Cao G (2008). Transforming growth factor-beta-regulated miR-24 promotes skeletal muscle differentiation.. Nucleic Acids Res.

[95] Wang Q, Huang Z, Xue H, Jin C, Ju XL (2008). MicroRNA miR-24 inhibits erythropoiesis by targeting activin type I receptor ALK4.. Blood.

[96] Mishra PJ, Humeniuk R, Longo-Sorbello GS, Banerjee D, Bertino JR (2007). A miR-24 microRNA binding-site polymorphism in dihydrofolate reductase gene leads to methotrexate resistance.. Proc Natl Acad Sci U S A.

[97] Cheng AM, Byrom MW, Shelton J, Ford LP (2005). Antisense inhibition of human miRNAs and indications for an involvement of miRNA in cell growth and apoptosis.. Nucleic Acids Res.

[98] Fearon ER, Vogelstein B (1990). A genetic model for colorectal tumorigenesis.. Cell.

[99] Maindonald J, Braun J (2007). Data Analysis and Graphics Using R..

[100] Storey JD, Tibshirani R (2003). Statistical significance for genomewide studies.. Proc Natl Acad Sci U S A.

[101] Thomson JM, Parker J, Perou CM, Hammond SM (2004). A custom microarray platform for analysis of microRNA gene expression.. Nat Methods.

