# Supplementary figures and images for: Pre-Micro RNA Signatures Delineate Stages of Endothelial Cell Transformation in Kaposi Sarcoma
Source: PLoS Pathog. 2009 Apr 17;5(4):e1000389. doi: 10.1371/journal.ppat.1000389 (PMC2663814; doi:10.1371/journal.ppat.1000389)

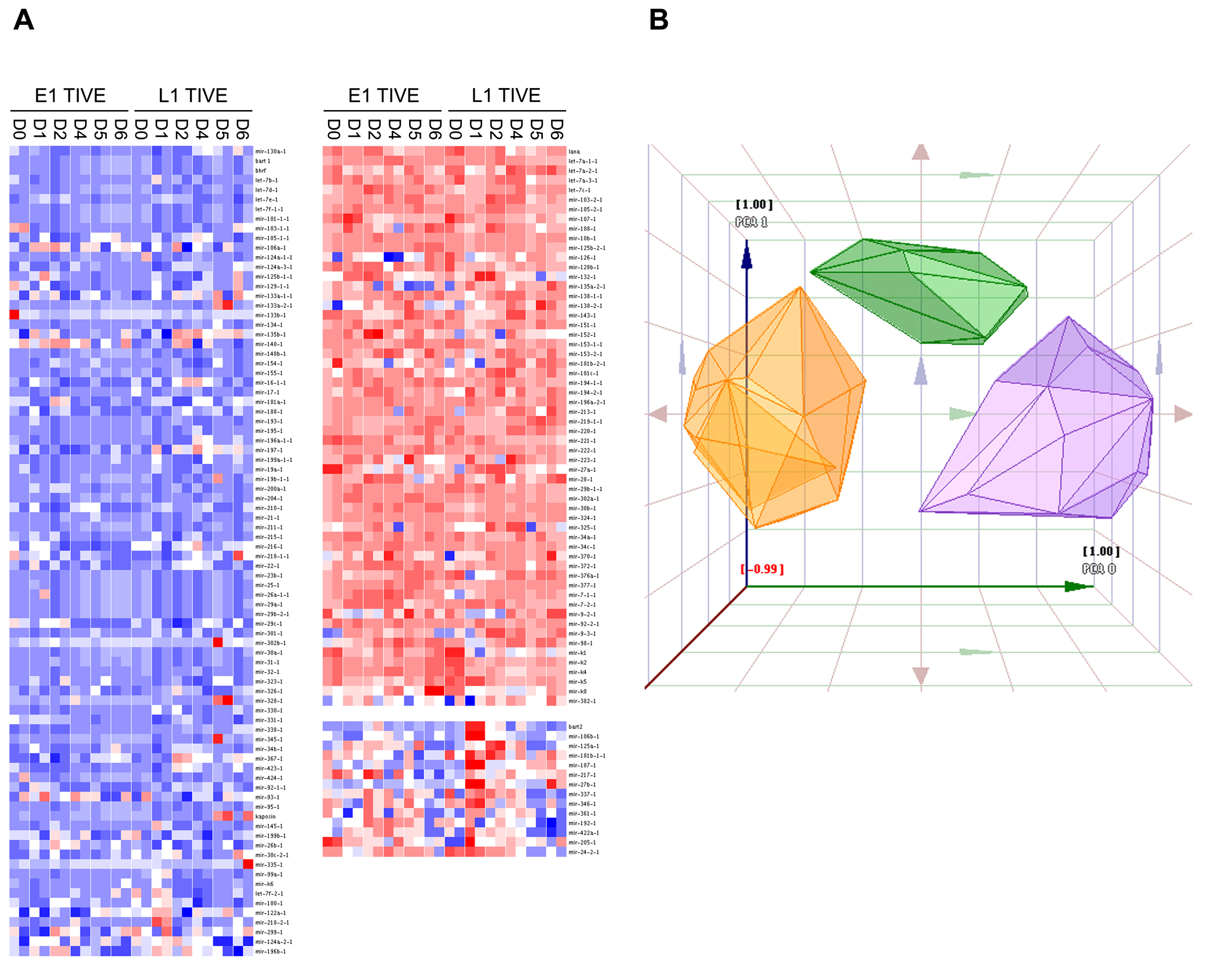

Supplement: Figure S1 — Minimal influence of proliferation phase on pre-miRNA levels in E1 and L1 cells. (A) Clustering analysis indicates few miRNAs changed in expression pattern during growth phases. (B) Principal component analysis of clustering indicates non-overlapping clusters. (1.57 MB TIF) [file ppat.1000389.s001.tif]

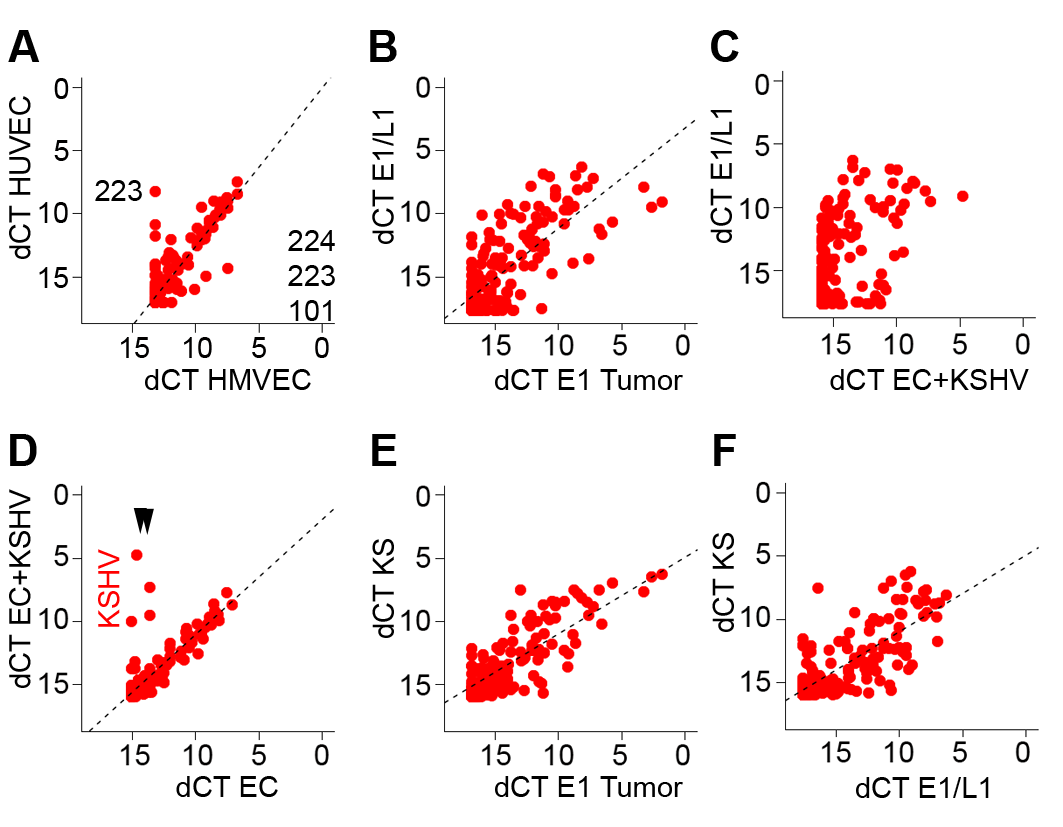

Supplement: Figure S2 — Pair-wise comparisons of dCT values for pre-miRNAs. (A) HUVEC against HMVEC, (B) KSHV-infected HUVEC against EC, (C) E1/L1 against KSHV-infected HUVEC, (D) E1/L1 cells in culture against E1 tumors, (E) KS biopsies against E1 tumors, (F) KS tumors against E1/L1 cell lines. Plotted are median dCTU6 values. (2.66 MB TIF) [file ppat.1000389.s002.tif]

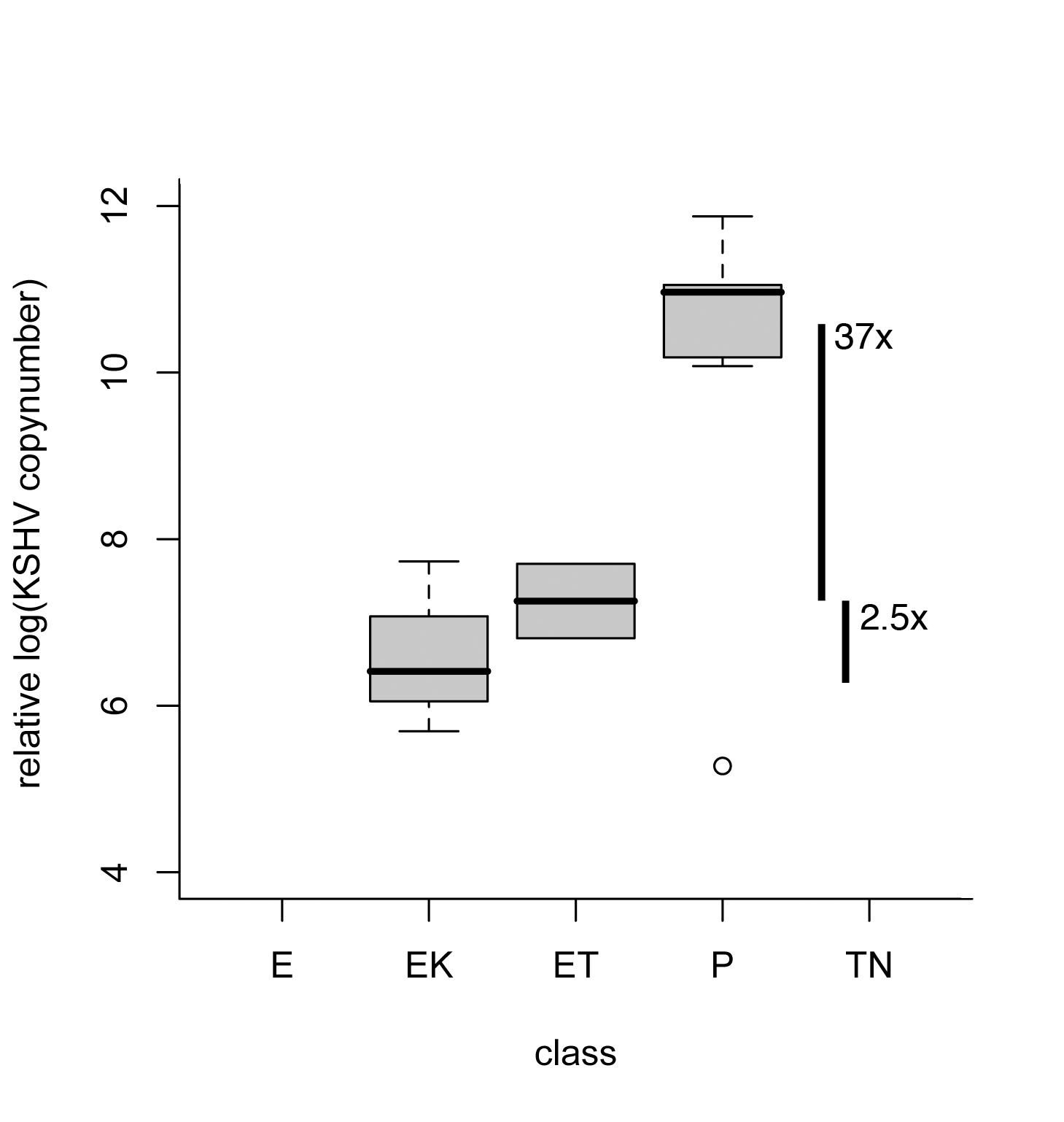

Supplement: Figure S3 — Relative KSHV genome copy number (2dCTU6) based on real-time QPCR for the LANA orf. The classes are (E) uninfected endothelial cells, (EK) KSHV infected endothelial cells, (ET) KSHV-infected endothelial cells, which can form tumors in nude mice (E1 and L1 TIVE), (P) Pel lines, (TN) tonsil. Shown also is the fold difference in relative copy number between KSHV-positive classes. Neither E nor RN yielded a detecable signal (1 copy per <100,000 cell equivalents). (2.26 MB TIF) [file ppat.1000389.s003.tif]

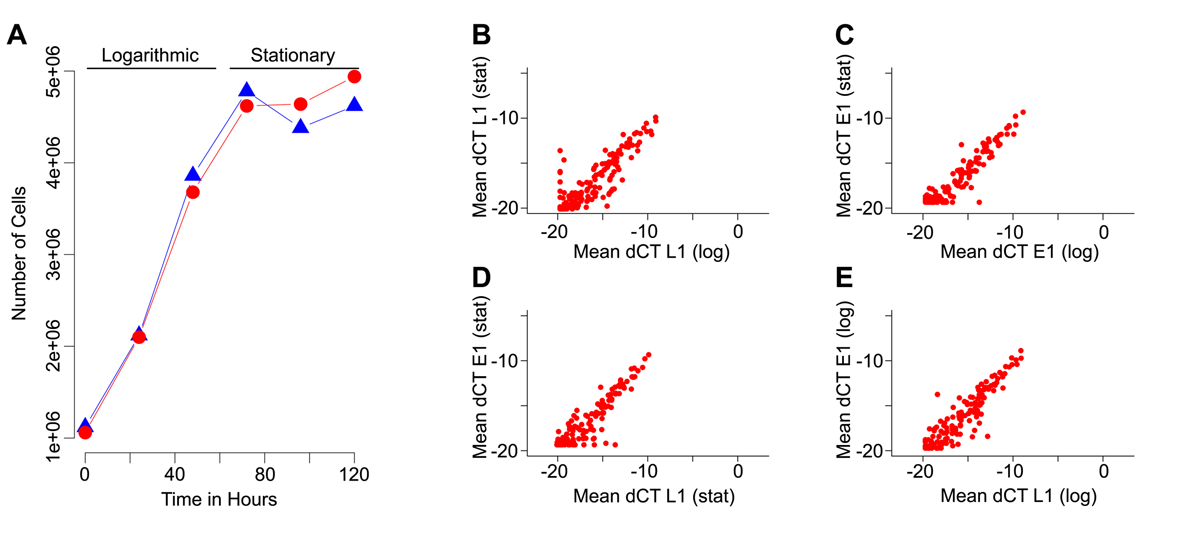

Supplement: Figure S4 — Pre-miRNA profiling throughout the cell cycle indicates few miRNAs are changed. (A) Growth curves for the E1 (red dots) and L1 (blue triangles) cell lines. Number of cells per T75 flask is shown on vertical axis and time in hours since seeding on the horizontal axis. Logarithmic indicates the time points that were averaged to yield logarithmic phase pre-miRNA levels and Stationary indicates time points that were averaged to yield stationary phase pre-miRNA levels. (B–E) Pair-wise comparisons of average log-pre-miRNA levels (dCTU6) of proliferation phases and cell lines. (Only data points with SD ≤3 were included in the comparison). (1.94 MB TIF) [file ppat.1000389.s004.tif]
