# Supplementary material for: Pre-Micro RNA Signatures Delineate Stages of Endothelial Cell Transformation in Kaposi Sarcoma
Source: PLoS Pathog. 2009 Apr 17;5(4):e1000389. doi: 10.1371/journal.ppat.1000389 (PMC2663814; doi:10.1371/journal.ppat.1000389)
Supplement: Table S1 — Indicated are the samples profiled, viral status and original cell line reference paper or sample source. (0.08 MB DOC) [file ppat.1000389.s005.doc]

**Supplemental Table S1:** Indicated are the samples profiled, viral status and original cell line reference paper or sample source.

| **Samples** | **Viral Status** | | **Reference** |  | **Samples** | **Viral Status** | | **Reference** |
| --- | --- | --- | --- | --- | --- | --- | --- | --- |
|  | KSHV | EBV |  |  |  | KSHV | EBV |  |
| BC1 | + | + | (Cesarman, 1995; Cesarman, 1995) |  | HUVEC KSHV C1 | + | - | (Wang, 2008) |
| BC2 | + | + | (Cesarman, 1995) |  | HUVEC KSHV C2 | + | - | (Wang, 2008) |
| BC5 | + | + | (Guasparri, 2004) |  | HMVEC KSHV | + | - | (Wang, 2008) |
| CRO-AP2 | + | + | (Carbone, 1997 ) |  |  |  |  |  |
| CRO-AP5 | + | + | (Carbone, 1998) |  | BJAB | - | - | (Menezes, 1975) |
| JSC1 | + | + | (Cannon, 2000) |  | BL8 | - | + | (Xia, 2008) |
| BC3 | + | - | (Arvanitakis, 1996) |  | BL5 | - | + | (Xia, 2008) |
| BCBL | + | - | (Komanduri, 1996) |  | DG75 | - | - | (Ben-Bassat, 1977) |
| BCLM | + | - | (Ghosh, 2003) |  |  |  |  |  |
| BCP1 | + | - | (Boshoff, 1998) |  | CEM | - | - | (Foley, 1965) |
| VG1 | + | - | (Brander, 2001) |  | Jurkat | - | - | (Schneider, 1977) |
| CRO-AP3 | + | - | (Carbone, 1998) |  |  |  |  |  |
| HHB2 | + | - | (Gradoville, 2000) |  | T012 | - | - | [[1]](#footnote-2)a |
| TY1 | + | - | (Katano, 1999) |  | T013 | - | - | a |
|  |  |  |  |  | T014 | - | - | a |
| BJAB+KSHV | + | - | (Nun, 2007) |  | T127 | - | - | a |
|  |  |  |  |  | T176 | - | - | a |
| SLK | - | - | (Herndier, 1994) |  | T179 | - | - | a |
| E1 TIVE | + | - | (An, 2006) |  | T200 | - | - | a |
| L1 TIVE | + | - | (An, 2006) |  | T202 | - | - | a |
|  |  |  |  |  | T262 | - | - | a |
| KS 101 | + | - | [[2]](#footnote-3)b |  |  |  |  |  |
| KS 165 | + | - | b |  | HUVEC | - | - | (Wang, 2008) |
| KS 299 | + | - | b |  | HMVEC | - | - | (Wang, 2008) |
| KS 310 | + | - | b |  |  |  |  |  |
| KS 322 | + | - | b |  |  |  |  |  |
| KS 336 | + | - | b |  |  |  |  |  |
| KS 352 | + | - | b |  |  |  |  |  |
| KS 4203 | + | - | [[3]](#footnote-4)c |  |  |  |  |  |
| KS 1315 | + | - | c |  |  |  |  |  |

1. ^a^ Obtained from Cooperative Human Tissue Network (CHTN) [↑](#footnote-ref-2)
2. ^b^ Obtained from collaborators at University of Miami [↑](#footnote-ref-3)
3. ^c^ Obtained from collaborators at Harvard University [↑](#footnote-ref-4)
